# Assessment of the diet of the critically endangered northern hairy‐nosed wombat (*Lasiorhinus krefftii*) using DNA metabarcoding

**DOI:** 10.1002/ece3.10469

**Published:** 2023-09-07

**Authors:** Fiona Casey, Julie M. Old, Hayley Jade Stannard

**Affiliations:** ^1^ School of Science Western Sydney University Hawkesbury New South Wales Australia; ^2^ School of Agricultural, Environmental and Veterinary Sciences Charles Sturt University Wagga Wagga New South Wales Australia

**Keywords:** dietary, grasses, herbivore, marsupial, seasonal, temporal

## Abstract

Invasive buffel grass (*Cenchrus ciliaris*) is considered a threat to the critically endangered northern hairy‐nosed wombat (*Lasiorhinus krefftii*; NHW). Buffel grass outcompetes native grasses, reducing availability of native food items for NHW, and causes more intense fires due to the large volumes of dead matter it produces. Previous studies suggested buffel grass was increasing in the diet; however, the diet of the NHW has not been reassessed for over two decades and was limited to Epping Forest National Park, with the population at Richard Underwood Nature Refuge having never been assessed. The recently released 2022 Recovery Action Plan for the species outlined objectives to assist its conservation and recommended the impact of buffel grass on the species' diet be investigated. This study aimed to determine: (1) which plant species are being consumed by the NHW; (2) the differences in the diet between sites; (3) differences between seasons; and (4) the abundance of buffel grass in the diet. The diet was assessed using DNA metabarcoding of scat samples collected from both sites from winter 2020 to spring 2021. Site and season significantly affected the diet of the NHW. Buffel grass dominated the diet and has increased in the diet since past assessments. The findings of this study will support population and habitat management of the critically endangered NHW. Enhanced knowledge of dietary items consumed at both sites will also assist efforts to locate additional sites suitable for translocation.

## Graphical abstract

Diet of the critically endangered northern hairy‐nosed wombat was assessed. They are generalist herbivores consuming mostly grasses followed by legumes. The northern hairy‐nosed wombat diet is significantly different across seasons and sites. The study contributes to our understanding of diet choice and will enable future identification of translocation sites that contain preferred food items.

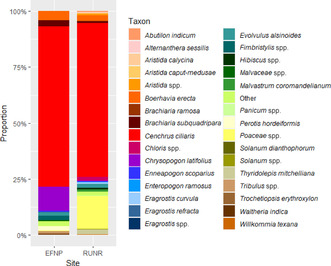

## 1 INTRODUCTION

The diet of a herbivore is determined by a combination of physiological and environmental factors, including food availability, gut morphology and nutritional requirements (Gordon & Illius, 1996; Illius & Gordon, 1992; Kohlmann & Rosenhoover, 1994). The diet of many herbivores, particularly large herbivores, is dominated by grasses, sedges, forbs and woody dicots (du Toit & Olff, 2014). Herbivores may consume a combination of dietary items that offer differing quantities of nutrients to fulfil specific requirements and achieve nutrient balance (Dussutour et al., 2010; Felton et al., 2009; Raubenheimer et al., 2005; Robbins et al., 2007; Rothman et al., 2011; Simpson et al., 2004). Although some factors that influence diet such as gut morphology cannot be manipulated, others such as food availability can, allowing conservationists to implement strategies to influence the diet of vulnerable or threatened species to improve their nutritional health. For herbivores, conservation strategies may involve valuable plant food items being promoted within their habitat while nondietary items may be controlled (Lindsay & Cunningham, 2011).

*Lasiorhinus krefftii*, known as the northern hairy‐nosed wombat (NHW), is a critically endangered (Taggart et al., 2016) herbivorous marsupial native to Australia. There are ~315 individuals living across two populations: ~300 at Epping Forest National Park (EFNP) and ~15 at Richard Underwood Nature Refuge (RUNR), a translocated population established in 2009 (Department of Environment and Science, 2021, 2022). The diet of the NHW at EFNP has been assessed in the past using histological techniques (Crossman, 1988; Flosser, 1986; Woolnough, 1998), but has not yet been assessed using DNA metabarcoding techniques, like the closely related southern hairy‐nosed wombat (*Lasiorhinus latifrons*; Sobek & Walker, 2021). Woolnough (1998) assessed the diet of the NHW using stable isotopes and long‐chain alkanes, but to minimise bias introduced by varying analyses, comparisons made throughout this study will refer to the results of histological analysis only. In this study, grass species constituted a large proportion of the diet, particularly *Heteropogon, Enneapogon* and *Aristida* species. Introduced *Cenchrus ciliaris*, otherwise known as buffel grass, was also identified as a dietary item. A variety of forbs complemented the diet, as well as the sedge *Fimbristylis dichotoma* (Woolnough, 1998). The diet of the NHW population at RUNR has never been assessed.

DNA metabarcoding is more specific and faster than traditional histological studies, allowing a greater number of samples to be processed in a shorter amount of time. It also allows for a lower resolution of taxonomic level to be identified, which is more difficult to determine using microscopic identification methods as some species are morphologically very similar at the species or Genus level, and can undergo changes due to digestion (Kaunisto et al., 2017; Valentini et al., 2009). DNA metabarcoding also is more likely to identify cryptic species, as these species are less likely to be chosen as reference material in histological studies (Thongtam na Ayudhaya et al., 2017).

Comparisons of previous NHW diet studies indicate that the diet has changed over time (Flosser, 1986; Woolnough, 1998) with some changes being linked to changes in forage availability and floristic composition due to severe droughts (Horsup, 1998). Other dietary changes, however, have been linked to the increase in abundance of buffel grass throughout EFNP (Horsup, 2004), where it is now the dominant grass species, occurring more frequently and contributing more to biomass than any other dietary item (Woolnough, 1998). The increase in buffel grass at EFNP was reported to have been followed by a dramatic increase in buffel grass in the diet of the NHW (Horsup, 2004). The abundance of buffel grass increased from ~1% in 1982–1983 (Flosser, 1986) to up to 27% during 1993–1996 (Woolnough, 1998). The occurrence of buffel grass within the floristic community at RUNR has also reportedly increased (Department of Environment and Science, 2022). This history of changes in the diet of the NHW suggests the diet may have again changed substantially since the most recent assessment over two decades ago.

The NHW Recovery Action Plan 2022 (Department of Environment and Science, 2022) outlined that one of four major objectives of the recovery plan is to fill gaps in our knowledge of the NHW; one knowledge gap identified as requiring investigation was the impact of buffel grass on the diet of the NHW. Buffel grass is considered a threat to the NHW as it has changed the composition of the vegetation community at both EFNP and RUNR, in turn changing the populations' food resources (Department of Environment and Science, 2022). Buffel grass grows and invades new habitats quickly and significantly outcompetes native Australian grasses, reducing overall species richness within an area (Marshall et al., 2012; Miller et al., 2010). Buffel grass causes hotter and faster fires because it produces large volumes of standing dead matter, more so than Australian native grasses. Soil disturbance caused by burrow building by NHW allows buffel grass to easily establish around burrows and outcompete native grasses, which in turn causes wombats to forage further from their burrow (and hence refuge) to find food (Marshall et al., 2012). An updated assessment of the current diet of the NHW will inform management decisions, including strategies such as controlling potentially threatening forage taxa, for example buffel grass or promoting native endemic food items, as previously described in Woolnough (1998). Improved access to food items of sufficient nutritional quality could support successful reproduction (Trites & Donnelly, 2003) and survival rates (Hutchings et al., 2003; Lochmiller & Deerenberg, 2000) of the NHW, thus supporting population growth.

This project aimed to determine the: (1) plant species being consumed by the NHW using modern DNA metabarcoding techniques, (2) differences in the diet across the two sites where NHW live, (3) seasonal changes to diet choice and (4) abundance of buffel grass in the diet.

## 2 METHODS

### 2.1 Study area

The two NHW population sites, EFNP and RUNR, were the focus areas for this study. EFNP is a 2750 hectare area of open eucalypt woodland within the Brigalow belt (22°21′ S and 146°41′ E), central Queensland (Department of Environment and Science, 2022). RUNR is a 130 hectare area of eucalypt woodland along river levees at Yarran Downs, southern Queensland (Department of Environment and Science, 2022).

### 2.2 Sample collection

Scat collection occurred across nine sampling periods. Scats were collected from EFNP during four seasons: winter and spring 2020, summer 2020/2021 and winter 2021; and from RUNR during five seasons: winter 2020, summer 2020/2021 and autumn, winter and spring 2021. Between eight and 11 scat samples were obtained during each sampling period (Appendix Table A1). There are approximately 300 NHW at EFNP and 15 at RUNR with 20 burrows recognised as ‘primary’ burrows frequently occupied by wombats at RUNR (Department of Environment and Science, 2022; Jørgensen et al., 2021). Except for four historic scat samples collected from EFNP in 2013, 2014, 2015 and 2017, all other scats were collected from near burrow entrances and all pellets were collected (as opposed to a single pellet from a group). One pellet was kept for assessment in this study, and the remainder were used for other research (Casey et al., unpub.). Scats were selected based on freshness as indicated by moistness (Banks et al., 2002), and effort was made to avoid contamination from substrates such as dirt (McInnes et al., 2017) and insects (dung beetles). The date, time and GPS coordinates for all scats collected, and the closet burrow number, were recorded. Scats were stored in paper bags at −80°C until analysis, on average scats were in the paper bag 2 h before being frozen.

### 2.3 DNA extraction, sequencing and bioinformatics

Scat samples were sent to the Australian Genome Research Facility in Adelaide, Australia. DNA was extracted using the DNeasy PowerSoil Pro DNA Extraction Kit (Qiagen) according to the manufacturer's instructions. Of 85 scat samples, 77 yielded DNA of adequate quality for analysis. The presence of plant DNA from faecal samples was detected using the primer pair ITS2F (TGTGAATTGCARRATYCMG) and ITS2R (CCCGHYTGAYYTGRGGTCDC; Moorhouse‐Gann et al., 2018) producing a 250 bp fragment. Image analysis was performed in real time by MiSeq Control Software version 3.1.0.13 and Real‐Time Analysis version 1.18.54.4. Sequencing was performed using an Illumina MiSeq platform (Ravi et al., 2018) and sequence data generated using the Illumina bcl2fastq 2.20 pipeline. Sterile water was run as a negative control.

The bioinformatics analysis consisted of demultiplexing, quality control, Amplicon Sequence Variant (ASV) calling and taxonomic classification. Diversity profiling analysis was performed with QIIME 22019.7 (Bolyen et al., 2019). Demultiplexed raw reads were primer trimmed and quality filtered using the cutadapt plugin, and denoising was performed with DADA2 (Callahan et al., 2016; via q2‐dada2). Taxonomy was assigned to ASVs with a 97% confidence threshold using the q2‐feature‐classifier (Bokulich et al., 2018) classify‐sklearn naïve Bayes taxonomy classifier.

### 2.4 Statistical analyses

The data were summarised as relative read abundance (RRA) and frequency of occurrence (FOO). RRA referred to the percentage of total reads a taxon contributes per sample and was used as an indication of the abundance of taxa in the diet in this study (Deagle et al., 2019). FOO referred to the percentage of samples in which a taxon was detected. A PERMANOVA was performed to detect whether the abundance of each taxon in the diet was affected by site and season. A zero‐adjusted Bray–Curtis dissimilarity index was calculated for each site, and an ANOSIM (analysis of similarities) was used to detect whether the diet varied between seasons at each site, with 9999 permutations of the test statistic. SIMPER (Similarity percentages) was used to determine the average dissimilarity between seasons at each site and how much of a contribution (%) each taxon made to the dissimilarity. Analyses were conducted using Primer 7 (Clark & Warwick, 2001).

## 3 RESULTS

Of the total 85 scats available for sequencing, we were unable to extract DNA from eight scats, including two collected from RUNR during winter 2020, two collected during winter 2020 and three collected during winter 2021 from EFNP, and the single scat collected from EFNP during 2015. Sequencing yielded a total of 9,984,833 reads from a total of 77 scat samples (Mean = 129,673 ± 34,717 S.D.). Sequencing yielded 4,709,816 reads from 39 scat samples from EFNP (Mean = 130,828 ± 33,692 S.D.) and 4,870,462 reads from 38 scat samples from RUNR (Mean = 128,170 ± 36,906 S.D.) during 2020–2021. Three of the four DNA samples extracted from scats collected from EFNP during 2013–2017 resulted in 139,659 sequences from 2013, 157,191 sequences from 2014 and 107,705 sequences from 2017. In total, 191 taxonomic categories were identified in the diet. At EFNP 20 orders, 32 families, 80 genera and 87 species, and at RUNR 20 orders, 33 families, 87 genera and 72 species of plant were identified in the scats.

### 3.1 Frequency and abundance

Overall *Poaceae* (grasses) contributed to 90.75% of RRA (across both sites) for the NHW. Four other plant families each contributed over 1% to diet abundance (RRA): *Nyctaginaceae* (3.07%), *Malvaceae* (1.83%), *Convolvulaceae* (1.47%) and *Cyperaceae* (1.05%). At the species level, buffel grass was the most abundant in the diet (68.93%), followed by unidentified *Poaceae* spp. (7.32%), *Chrysopogon latifolius* (5.99%) and *Boerhavia erecta* (3.07%). Five other species contributed >1% to total diet abundance. Unassigned spp. at any taxonomic level contributed <1% to diet abundance but had a FOO of 94% (was detected in 72 of 77 samples). Buffel grass was the only taxon with a FOO of 100%. The FOO of *Poaceae* spp. *Chrysopogon latifolius* and *Boerhavia erecta* was 82%, 61% and 62%, respectively.

### 3.2 Site differences

**FIGURE 1:**
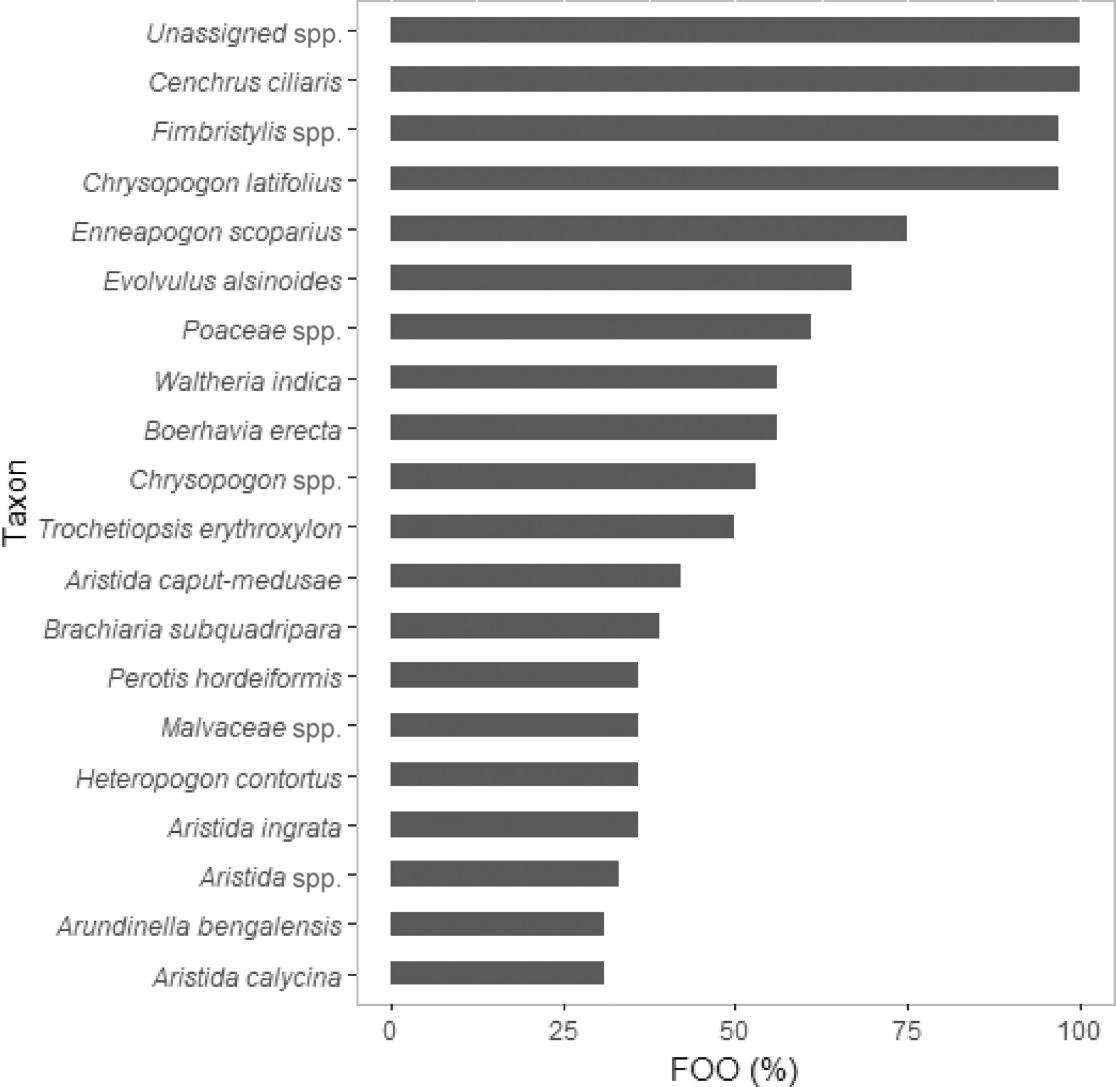
Frequency of occurrence (FOO) of taxa in the diet of northern hairy‐nosed wombat at Epping Forest National Park during 2020–2021. Only taxa with a FOO of ≥30% are represented.

**FIGURE 2:**
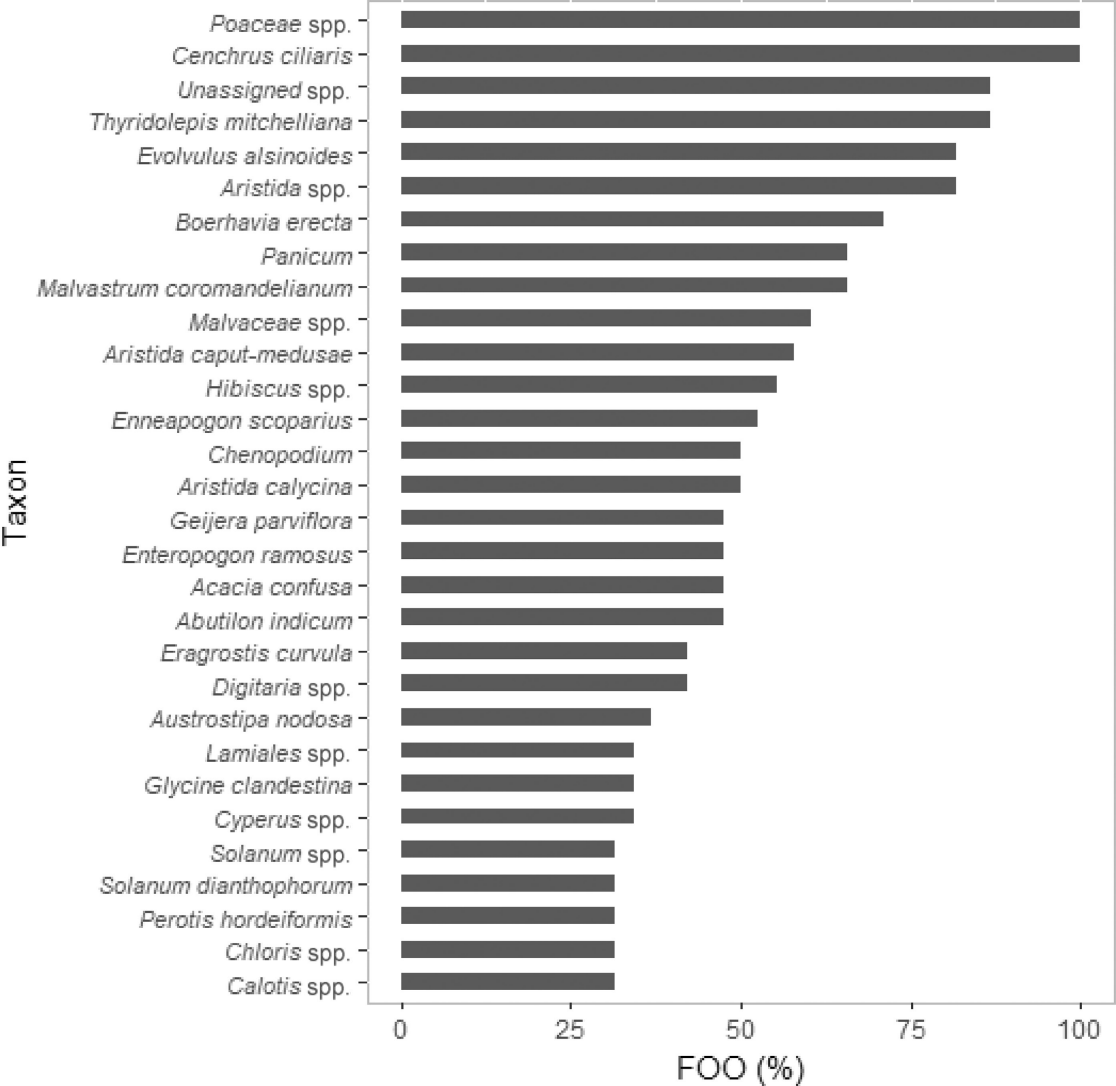
Frequency of occurrence (FOO) of taxa in the diet of northern hairy‐nosed wombat at Richard Underwood Nature Refuge during 2020–2021. Only taxa with a FOO of ≥30% are represented.

Of the 191 taxonomic categories identified within the diet, 71 were present in the diet at both sites, 64 were unique to EFNP, and 56 were unique to RUNR. For nine taxa, the difference in FOO between EFNP and RUNR was <1%. For example, *Fungi* spp., *Trichoderma* spp., *Cystobasidiaceae* spp. and *Chloris pumilio* each had a FOO of 2.8% at EFNP and 2.6% at RUNR. For seven taxa, the difference in FOO between EFNP and RUNR was >50%. The FOO of *Fimbristylis* spp., *Chrysopogon latifolius* and *Waltheria indica* was, respectively, 87%, 74% and 56% greater at EFNP compared with RUNR (Figures 1 and 2). The FOO of *Thyridolepis mitchelliana*, *Panicum* spp., *Malvastrum coromandelianum* and *Chenopodium* spp., was, respectively, 50%, 52%, 66% and 84% greater at RUNR than EFNP (Figures 1 and 2).

**FIGURE 3:**
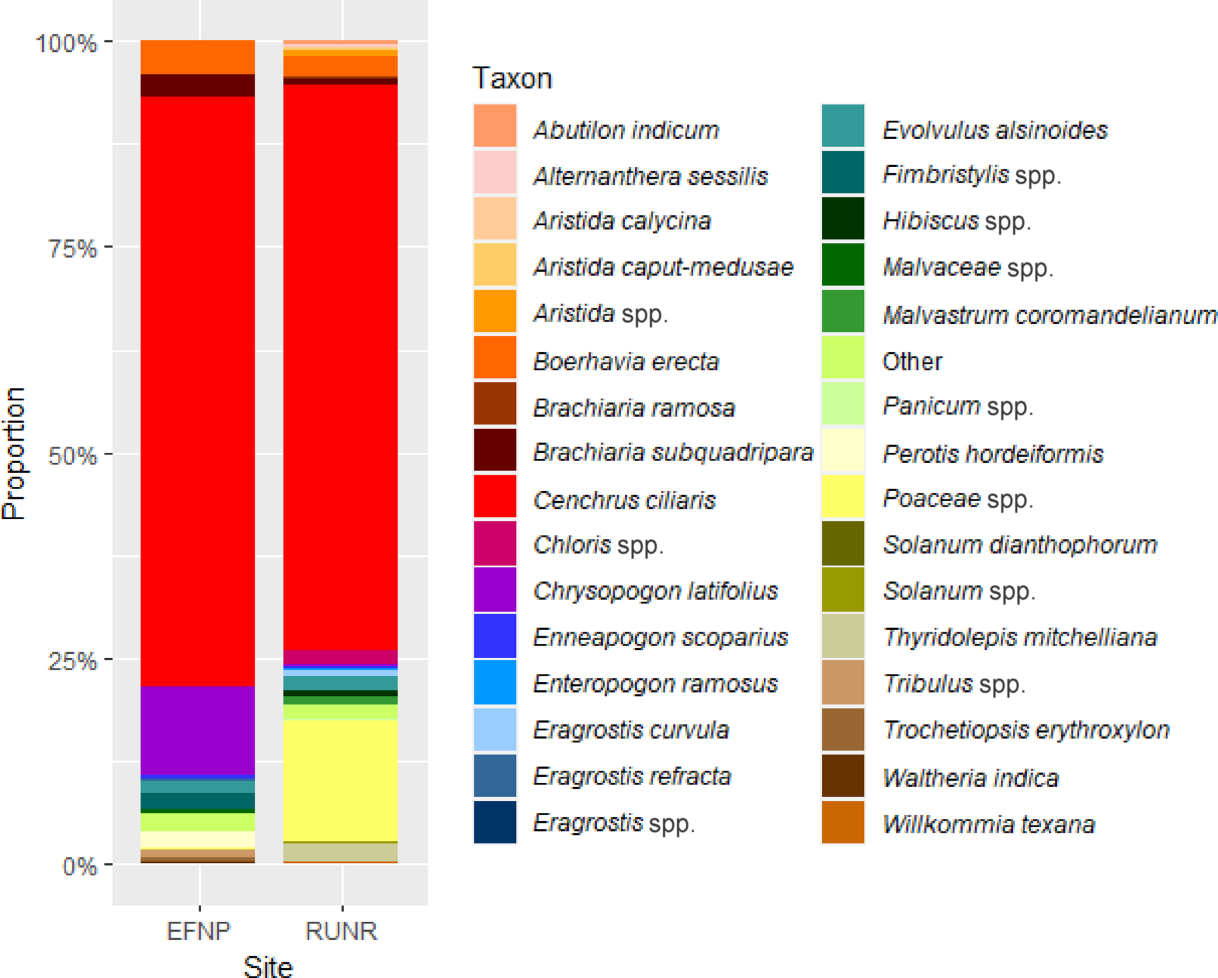
Proportional abundance of taxa in the diet of northern hairy‐nosed wombat at Epping Forest National Park (EFNP) and Richard Underwood Nature Refuge (RUNR) during 2020–2021. All taxa that did not contribute ≥0.5% within at least one season are combined and represented as ‘Other’.

At EFNP, eight taxa returned an abundance of ≥0.5%, including unidentified *Tribulus* spp. (Figure 3) which was not present in the diet at RUNR. Twelve taxa returned an abundance of ≥0.5% at RUNR, including *Eragrostis curvula* and unidentified *Chloris* spp. which were not present in the diet at EFNP. Four taxa contributed ≥0.5% at both sites: buffel grass, *Boerhavia erecta*, *Evolvulus alsinoides* and *Brachiaria subquadripara*. Buffel grass was the most abundant taxon in the diet at both sites; the average abundance of buffel grass in the diet at EFNP was 71.6%, and 68.6% at RUNR (Figure 3).

The PERMANOVA revealed that there was a significant interaction between site and season affecting the abundance of items within the diet (*p ≤* .05). The pairwise SIMPER revealed the two greatest contributors to dissimilarity between the two sites were the mean abundance of buffel grass, which was greater at EFNP compared with RUNR, followed by the mean abundance of unidentified *Poaceae* spp., which was greater at RUNR compared with EFNP (Figure 3). The next six greatest contributors to dissimilarity between the two sites were *Chrysopogon latifolius, Boerhavia erecta, Brachiaria subquadripara, Perotis hordeiformis*, unidentified species of the *Fimbristylis* genus and unidentified species of the *Tribulus* genus, which were all more abundant in the diet at EFNP compared with RUNR (Figure 3).

### 3.3 Seasonal differences at EFNP

**FIGURE 4:**
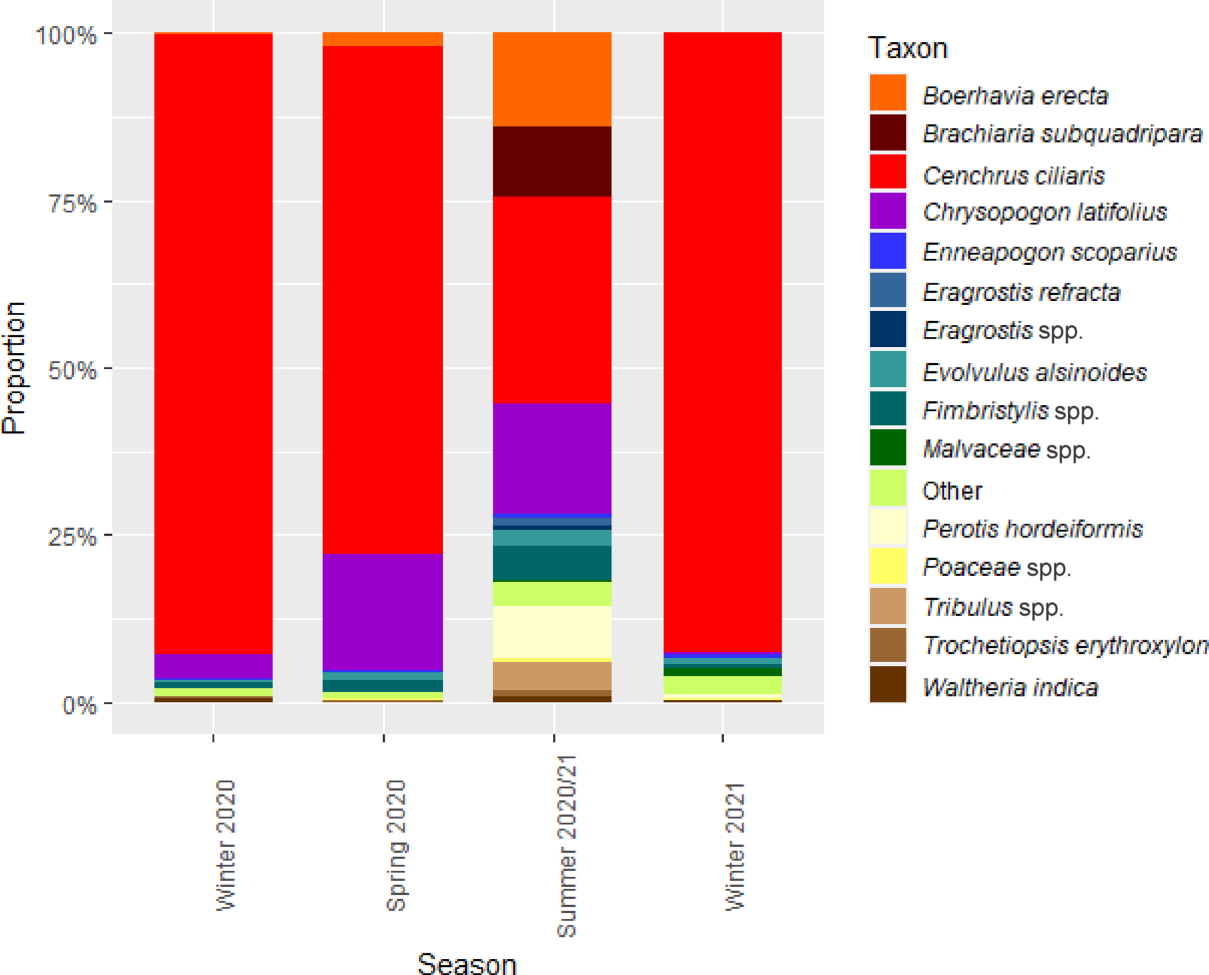
Proportional abundance of taxa in the diet of northern hairy‐nosed wombat at Epping Forest National Park, in total and across each season. All taxa that did not contribute ≥0.5% within at least one season are combined and represented as ‘Other’.

At EFNP, buffel grass returned the highest abundance of all taxa during every season; the average abundance of buffel grass was greater during winter 2020 (92.6%) and winter 2021 (92.5%) compared with spring 2020 (76.0%) and summer 2020/2021 (30.8%; Figure 4). *Chrysopogon latifolius* was the second most abundant taxon within the diet during winter 2020 (3.7%), followed by unidentified *Fimbristylis* spp. (1.0%). *Chrysopogon latifolius* returned the second highest abundance during spring 2020 (17.5%) and summer 2020/2021 (16.5%), followed by *Boerhavia erecta* (spring 2020 = 2.0%; summer 2020/2021 = 13.9%). During winter 2021, unidentified *Malvaceae* spp. returned the second highest abundance within the diet (1.2%), followed by *Evolvulus alsinoides* (1.0%; Figure 4).

At EFNP, two taxa had a FOO of 100% in every season: buffel grass and unassigned spp. Buffel grass, *Chrysopogon latifolius*, *Fimbristylis* spp. and unassigned spp. were the only taxa with a FOO of 100% during winter 2020 and spring 2020. Despite returning the third highest abundance, the FOO of *Boerhavia erecta* during spring 2020 was 33%. During summer 2020/2021, 11 taxa had a FOO of 100%, including *Boerhavia erecta*, *Chrysopogon latifolius* and *Fimbristylis* spp. (Appendix Table A2). Buffel grass and unassigned spp. were the only two taxa with a FOO of 100% during winter 2021, while the FOO of unidentified *Malvaceae* spp. and *Evolvulus alsinoides* was 86% and 63%, respectively (Appendix Table A2).

The one‐way ANOSIM revealed that there were significant differences between seasons at EFNP (*p* ≤ .05), with pairwise tests revealing significant differences between winter 2020 and summer 2020/2021 (*p ≤* .05), spring 2020 and summer 2020/2021 (*p ≤* .05), and summer 2020/2021 and winter 2021 (*p ≤* .05). The pairwise SIMPER revealed an average dissimilarity of 66.79 between summer 2020/2021 and winter 2021, 64.53 between winter 2020 and summer 2020/2021 and 57.72 between spring 2020 and summer 2020/2021 at EFNP (Table 1). Within those three significantly different pairwise comparisons, buffel grass, *Chrysopogon latifolius* and *Boerhavia erecta* were the three highest contributors to dissimilarity (Table 1).

**TABLE 1:** Pairwise SIMPER results including the 10 taxa with the greatest contribution to dissimilarity for significant pairwise ANOSIM tests from Epping Forest National Park (EFNP).

Pairwise text	Winter 20 and summer		Spring 20 and summer		Summer and winter 21	
Pairwise test: Av. Diss	64.53		57.72		66.79	
Taxa	Av. diss	Cont. (%)	Av. diss	Cont. (%)	Av. diss	Cont. (%)
*Boerhavia erecta*	6.84	10.60	6.63	11.49	6.93	10.38
*Brachiaria subquadripara*	5.30	8.21	5.30	9.18	5.33	7.97
*Cenchrus ciliaris*	30.88	47.85	22.58	39.12	30.84	46.17
*Chrysopogon latifolius*	7.48	11.59	9.19	15.93	8.13	12.17
*Eragrostis refracta*	0.66	1.02	0.66	1.14	1.35	0.99
*Evolvulus alsinoides*	1.20	1.86	1.56	2.70	2.16	2.02
*Fimbristylis* spp.	2.00	3.09	1.76	3.05	2.23	3.35
*Malvaceae* spp.					0.66	0.90
*Perotis hordeiformis*	3.86	5.99	3.85	6.67	3.70	5.54
*Tribulus* spp.	2.16	3.35	2.16	3.73	2.16	3.23
*Waltheria indica*	0.48	0.75	0.44	0.77		

Abbreviations: Av. diss, average dissimilarity; Cont (%), percentage contribution to dissimilarity.

The one‐way ANOSIM pairwise tests revealed that there was no significant difference in the diet between 2013, 2014, 2017 and any other sampling period at EFNP (*p* = .07–.50). However, the mean abundance of buffel grass in the diet increased from 40.8% during 2013–2017 to 71.6% during 2020/2021 (Table 2). The abundance of *Chrysopogon latifolius* in the diet halved from ~22% in 2013–2017 to ~11% in 2020–2021, and the abundance of *Enneapogon scoparius* and *Heteropogon contortus* decreased from ~10% in 2013–2017 to <0.5% in 2020–2021 (Table 2).

**TABLE 2:** Percentage mean abundance of each taxon in the diet of northern hairy–nosed wombat (NHW) at Epping Forest National Park (EFNP) during 2013–2017 compared to during 2020–2021 (including only taxa with mean abundance ≥0.5% during 2013–2017 or 2020–2021).

Taxon	Mean (%) 2013–2017	Mean (%) 2020–2021
	*n* = 3	*n* = 36
*Cenchrus ciliaris*	40.8	71.6
*Chrysopogon latifolius*	22.1	10.7
*Enneapogon scoparius*	10.5	0.4
*Heteropogon contortus*	9.2	0.2
*Brachiaria subquadripara*	7.2	2.7
*Melinis repens*	2.6	<0.1
*Perotis hordeiformis*	1.8	2.0
*Poaceae* spp.	1.0	0.3
*Fimbristylis* spp.	0.8	2.1
*Tribulus* spp.	0.1	1.1
*Boerhavia erecta*	0.1	4.2
*Evolvulus alsinoides*	<0.1	1.3

### 3.4 Seasonal differences at RUNR

**FIGURE 5:**
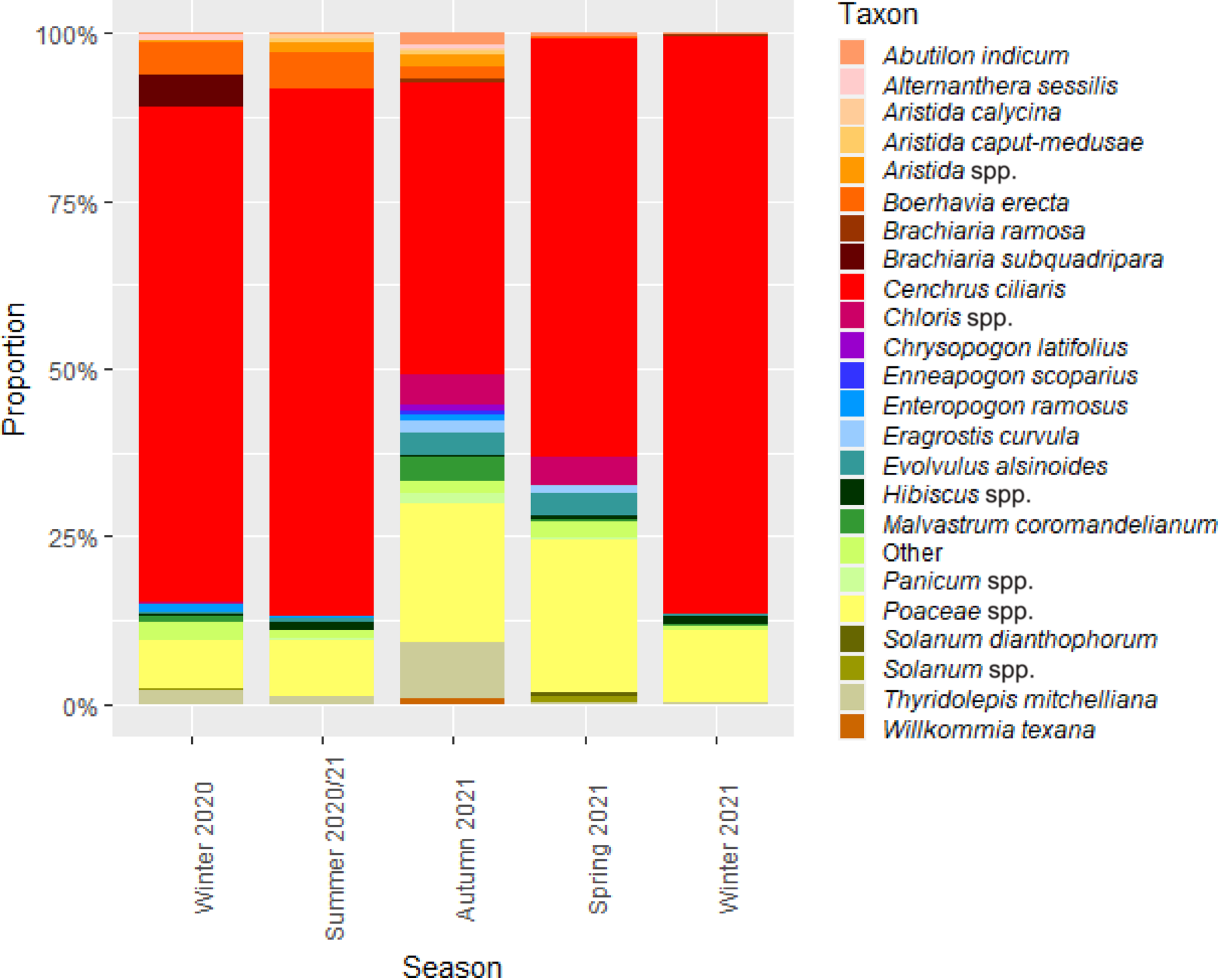
Proportional abundance of taxa in the diet of northern hairy‐nosed wombat at Richard Underwood Nature Refuge, in total and across each season. All taxa that did not contribute ≥0.5% within at least one season are combined and represented as ‘Other’.

At RUNR, buffel grass was the most abundant taxon within the diet during every season; the abundance of buffel grass was greatest during winter 2021 (86.1%) and lowest during autumn 2021 (43.4%; Figure 5). Unidentified *Poaceae* spp. was the second most abundant taxon within the diet during every season: the abundance of unidentified *Poaceae* spp. was greatest during spring 2021 (22.8%) and lowest during winter 2020 (7.4%; Figure 5). Following buffel grass and *Poaceae* spp., the next most abundant taxon within the diet during each season was *Boerhavia erecta* during winter 2020 (4.9%) and summer 2020/2021 (5.2%), *Thyridolepis mitchelliana* during autumn 2021 (8.2%), unidentified *Hibiscus* spp. during winter 2021 (1.4%) and unidentified *Chloris* spp. during spring 2021 (4.1%; Figure 5).

Buffel grass and unidentified *Poaceae* spp. were the only taxa with a FOO of 100% during every season at RUNR (Appendix Table A3). Seven taxa had a FOO of 100% during winter 2020, including *Boerhavia erecta* and *Thyridolepis mitchelliana*. Four taxa had a FOO of 100% during summer 2020/2021, including *Boerhavia erecta*, and eight taxa had a FOO of 100% during Autumn 2021, again including *Boerhavia erecta* and *Thyridolepis mitchelliana*. Buffel grass, *Poaceae* spp. and unassigned spp. were the only three taxa with a 100% FOO during winter 2021; *Hibiscus micranthus* had the next highest FOO of 75%, followed by seven taxa with a FOO of 50%. Five taxa had a FOO of 100% during spring 2021, including *Thyridolepis mitchelliana* (Appendix Table A3).

At RUNR, the one‐way ANOSIM among seasons revealed a significant effect of season on the diet (*R* = 0.305, *p* ≤ .05), with the pairwise tests revealing significant differences between all seasons (*R* = 0.232–0.560, *p* ≤ .05), except between summer 2020/2021 and winter 2020, and summer 2020/2021 and winter 2021. The pairwise SIMPER revealed the pairwise comparisons with the four highest average dissimilarities all involved autumn 2021, and within all significant pairwise tests at RUNR, buffel grass and *Poaceae* spp. were the two greatest contributors to dissimilarity (Table 3).

**TABLE 3:** Pairwise SIMPER results including the 10 taxa with the greatest contribution to dissimilarity for significant pairwise ANOSIM tests from Richard Underwood Nature Refuge (RUNR).

Pairwise text	Win 20 and Aut 21		Win 20 and win 21		Win 20 and Spr 21		Sum and Aut 21		Sum and Spr 21		Aut 21 and win 21		Aut 21 and Spr 21		Win 21 and Spr 21	
Pairwise test: Av. diss	45.87		22.37		30.73		46.4		30.81		47.23		38.61		27.6	
Taxa	Av. diss	Cont. (%)	Av. diss	Cont. (%)	Av. diss	Cont. (%)	Av. diss	Cont. (%)	Av. diss	Cont. (%)	Av. diss	Cont. (%)	Av. diss	Cont. (%)	Av. diss	Cont. (%)
*Abutilon indicum*	0.85	1.85					0.84	1.81			0.84	1.78	0.92	2.38		
*Alternanthera sessilis*			0.37	1.63												
*Aristida* spp.							1.06	2.29	1.47	2.65	0.81	1.73	0.79	2.05		
*Boerhavia erecta*	2.05	4.48	2.43	10.85	2.38	7.74	2.5	5.38	2.57	8.33	0.86	1.83	0.83	2.15		
*Brachiaria subquadripara*	2.34	5.1	2.37	10.59	2.37	7.71										
*Cenchrus ciliaris*	16.09	35.07	8	35.75	7.8	25.37	18.23	39.29	10.5	34.07	21.61	45.76	11.88	30.78	12.6	45.65
*Chloris* spp.	2.27	4.95			2.01	6.55	2.24	4.83	2.05	6.66	2.24	4.75	3.34	8.64	2.05	7.43
*Enteropogon ramosus*			0.55	2.46	0.55	1.77										
*Eragrostis curvula*	0.84	1.83			0.63	2.06	0.85	1.84	0.64	2.07	0.84	1.78	1.2	3.1	0.64	2.31
*Evolvulus alsinoides*	1.63	3.55	0.26	1.15	1.55	5.03	1.58	3.41	1.47	4.78	1.67	3.54	1.72	4.44	1.56	5.67
*Hibiscus* spp.			0.75	3.33					0.67	2.19					0.78	2.81
*Malvaceae* spp.															0.24	0.85
*Malvastrum coromandelianum*	1.97	4.3	0.53	2.38	0.56	1.82	1.8	3.88			1.77	3.74	1.81	4.7	0.27	0.97
*Poaceae* spp.	7.64	16.65	4.21	18.83	7.99	26.01	7.74	16.68	7.9	25.66	7.19	15.23	6.07	15.72	7.02	25.42
*Solanum dianthophorum*															0.25	0.9
*Solanum* spp.									0.46	1.49					0.46	1.66
*Thyridolepis mitchelliana*	3.53	7.69	1.01	4.51	0.96	3.12	3.73	8.03	0.43	1.41	4.06	8.6	3.97	10.27		

Abbreviations: Aut, autumn; Av. diss, average dissimilarity; Cont (%), percentage contribution to dissimilarity; Spr, spring; Sum, summer; Win, winter.

## 4 DISCUSSION

The diet of the NHW varied significantly seasonally and between EFNP and RUNR. Buffel grass dominated the diet of the NHW at both sites. Only eight taxa at EFNP and 12 taxa at RUNR contributed more than ≥0.5% each to the diet (Figure 3). There was a high number of taxa detected within the diet (191 taxa) but few primary dietary items, with the vast majority contributing <0.5% to dietary abundance (93% of taxa) and having a FOO <20% (84% of taxa). The taxa that did contribute ≥0.5% to dietary abundance often also had a higher FOO than most other taxa. Buffel grass heavily dominated the diet of the NHW, with a FOO of 100% and an abundance almost 10 times greater than the next most abundant taxon.

Despite buffel grass dominating the diet at both sites, there was significant difference in the diet of the NHW between EFNP and RUNR. Approximately two thirds of the taxa identified were present in the diet at one site but not the other, including some primary dietary items (contributed ≥0.5% to diet abundance within a site), such as *Tribulus* spp., *Eragrostis curvula* and *Chloris* spp. In addition, numerous taxa were primary dietary items at one site, but were of low abundance in the diet at the other, including *Chrysopogon latifolius* and unidentified *Poaceae* spp. (Appendix Tables A4 and A5). These differences in primary dietary items between EFNP and RUNR may be due to differences in the availability (frequency or biomass) of each taxon at each site. Previous studies at Epping Forest National Park have shown that buffel grass is the dominant grass species followed by *Aristida* spp., *Enneapogon* spp., *Eragrostris lacunaria*, *Chrysopogon fallax* and the sedge *Fimbristylis dichotoma*. There are temporal fluctuations in FOO, and long‐term drought has affected FOO of some species (Woolnough, 1998). Buffel grass increased in FOO from 8% to 48% from 1987 to 1997 and in biomass from 13% to 54% from 1987 to 1994 (Back, 1997; Horsup, 1998; Woolnough, 1998). RUNR is dominated by buffel grass and *Aristida* spp. and windmill grass (*Enteropogon ramosus*; Jørgensen et al., 2021).

Significant seasonal differences were found in the diet of the NHW at both EFNP and RUNR. Only one study has previously investigated temporal changes in the diet of the NHW. Woolnough (1998) assessed the diet of the NHW at EFNP and found the diet remained relatively consistent throughout the year, with no clear changes in composition between seasons. However, Woolnough (1998) recognised that the methods adopted to quantify the diet were likely not adequate for detecting subtle seasonal changes.

At EFNP, all seasonal pairwise comparisons were significant except between the two winter seasons (2020 and 2021) and between spring 2020 and each winter season (2020 and 2021). Like EFNP, every seasonal pairwise comparison was significant at RUNR except summer 2020/2021 and each winter season (2020 and 2021). Changes in forage availability within each site could be responsible for seasonal changes in the diet. The vegetation community at RUNR has been briefly described (Department of Environment and Science, 2018; Jørgensen et al., 2021), but no information regarding season or seasonal changes was provided. An assessment of pasture productivity at EFNP approximately three decades ago found productivity was low during 9 months of the year, over spring, winter and autumn, followed by a drastic increase of up to 20‐fold during summer (Johnson, 1991). Determining the influence of forage availability on the diet would require the forage community to be assessed alongside an assessment of the diet.

The results of this study revealed that buffel grass dominated the diet of the NHW, and as Woolnough (1998) suggested is likely a reflection of availability of the species within the habitat. Forage availability has also been found to influence diet choice in other herbivorous species (Hopcraft et al., 2010; McNaughton & Banyikwa, 1995); for example, elephants (*Loxodonta africana*) increase the amount of grasses consumed seasonally as grass biomass increases within the habitat (Shannon et al., 2013). The forage communities at EFNP and RUNR were not assessed in this study, but if forage availability is determining the diet of the NHW, the dominance of buffel grass in the diet would suggest that buffel grass is readily available throughout each site. An abundance of buffel grass in the habitat is a concerning possibility, as the invasive species has been identified as a threat to native vegetation communities within NHW habitat (Department of Environment and Science, 2022), and its impact on the nutrition of the species is currently unknown.

The diet of the NHW at EFNP had not significantly differed between samples collected in past years (2013–2017) and samples collected for the current study during 2020–2021; however, this result is unreliable as only a single sample represented each past year (2013, 2014 & 2017). Although the diet in past years was not significantly different, changes in the mean abundance of some dietary taxa, including buffel grass, may indicate narrowing dietary niche breadth of the NHW at EFNP. For example, the mean abundance of buffel grass increased more than 30% from 2013–2017 to 2020–2021, while the mean abundance of three taxa decreased by approximately 10% (Table 2). It should be noted only three scat samples were available for analysis from 2013 to 2017; hence, a larger sample size would have provided a better indication of changes and trends in the diet.

The diet of the NHW at EFNP has changed considerably to that reported in past assessments. Flosser (1986) assessed the species composition (%) of the diet and listed five taxa as primary dietary items: *Eragrostris lacunaria, Chrysopogon fallax, Heteropogon contortus, Fimbristylis* spp. and *Aristida* spp. Crossman (1988) assessed species frequency within the diet (%) and identified *Heteropogon contortus, Aristida* spp., *Enneapogon* spp. and *Sporobolus caroli* as primary dietary items. Woolnough (1998) was the first to report buffel grass as a primary dietary item, alongside *Aristida* spp. and *Enneapogon* spp. (Table 4). The contribution of buffel grass to the diet of the NHW at EFNP has been increasing over recent decades. Buffel grass contributed <1% to the diet up to 1986 (Flosser, 1986), then increased to ~14% by 1998 (Woolnough, 1998) and increased again to ~60% in our study (Table 4). The FOO of buffel grass in scats has increased from 20% in 1988 (Crossman, 1988) to 100% in 2020–2021. The contribution to the diet of species from the genera *Chrysopogon, Boerhavia, Brachiaria, Perotis* and *Tribulus* have also increased from previous assessments to 2020–2021 (Table 4). *Eragrostis* was the most dominant dietary item during 1986, contributing to ~14% of the diet (Flosser, 1986), yet contributed just 1% during 2020–2021, while *Aristida* spp. and *Enneapogon* spp. were the most dominant taxa within the diet during 1998, contributing ~21% and ~17%, respectively (Woolnough, 1998), yet contributed <1% each during 2020–2021 (Table 4). Hence, our study has shown there have been temporal changes to the diet of NHW since 1986.

**TABLE 4:** Abundance and frequency of occurrence (FOO) of each genus in the diet of the NHW at EFNP across four studies: Crossman (1988), Flosser (1986), and Woolnough (1998) and the current study. The genera included are those that contributed ≥1% to diet abundance in either Flosser (1986) and Woolnough (1998) or the current study, and every genera reported by Crossman (1988).

Genus	Flosser (1986)	Woolnough (1998)	Current study (2021)	Crossman (1988)	Current study (2021)
	Abundance (%)	Abundance (%)	Abundance (%)	FOO (%)	FOO (%)
*Aristida*	4.29	20.64	0.43	55	88
*Boerhavia*			7.34		
*Brachiaria*	1.11	0.01	5.64		
*Cenchrus*	0.89	14.46	59.84	20	100
*Chrysopogon*	5.37	0.04	8.94		
*Enneapogon*	1.74	17.21	0.66	50	88
*Enteropogon*	1.23	0.12	0.00	25	12
*Eragrostis*	14.14	0.04	1.00		
*Evolvulus*		1.85	1.80		
*Fimbristylis*	5.46	1.85	2.92	10	94
*Heteropogon*	3.97	0.00	0.30	80	41
*Panicum*				5	
*Perotis*	1.46	0.02	4.27		
*Sporobolus*				50	12
*Tribulus*		0.05	2.50		
Unidentified	58.37	40.34	5.76		
*Waltheria*		1.96	0.55		

When comparing the diet of the NHW over time, it is important to note that different dietary assessment techniques were used. Previously, histological techniques were used to assess the diet of the NHW (Crossman, 1988; Flosser, 1986; Woolnough, 1998) whereas the current study used DNA metabarcoding. The metabarcoding approach is more comprehensive than a histological approach, with improved capability for accurately detecting dietary items that are rare or consumed in very small quantities (Nichols et al., 2016; Soininen et al., 2009; Stapleton et al., 2022). For example, our metabarcoding technique identified more taxa in the diet compared with past assessments. Some taxa detected within the diet in this study, but not previously, may have been present in the diet at the time of previous assessments but in amounts not detectable by histological analysis. Additional taxa may also be identified in the future due to the expectation that increased sequence data for more plant species will increase in available databases over time. Although bias therefore exists in the comparison of past and current results, the variations in primary dietary items over time are pronounced and thus likely do reflect true changes in the diet.

Woolnough and Johnson (2000) found eastern grey kangaroos (*Macropus giganteus*) and NHW shared 85% of diet species. Sobek and Walker (2021) used DNA metabarcoding to compare the diet of the southern hairy‐nosed wombat and western grey kangaroo (*Macropus fuliginosus*) and found 20 genera and 10 genera, respectively, with eight genera in common. Both diets included introduced and native genera. In contrast to the southern hairy‐nosed wombat (Sobek & Walker, 2021), NHW consumed species in the genera *Austrostipa* (native), *Chenopodium* (native), *Euphorbia* (native), *Mediocargo* (introduced) and *Oxalis* (introduced). The highest occurrence of plants in southern hairy‐nosed wombat scats was from the *Atriplex, Carrichtera, Medicargo* and *Sclerolaena* genera (Sobek & Walker, 2021). In the NHW diet, the introduced *Oxalis corniculata* was the only species identified that was also in the diet of the southern hairy‐nosed wombat. Differences in wombat species distribution and floristic composition within the habitats likely account for differences in the diets of the two wombat species.

A limitation of this study was the use of RRA to quantify the abundance of each taxon within the diet. Using RRA to quantifying dietary abundance has been criticised for lacking accuracy, as several biological and technical factors can affect the number of sequence reads produced for each food item (Pompanon et al., 2012); however, the method has been recognised as capable of providing estimates (Deagle et al., 2019; Stapleton et al., 2022; Verkuil et al., 2022). Given buffel grass was detected in every sample and returned a mean RRA much greater than any other taxa, it is likely accurate that buffel grass does dominate the diet of the NHW at both sites.

Another limitation of this study is that the assessment of the diet of the NHW at EFNP is incomplete, as scat samples were not collected during autumn. Scats from all four seasons must be analysed to achieve representation of the whole diet, especially considering season was found to significantly affect the diet at EFNP. Aside from assessing the diet of the NHW at EFNP during all four seasons, the dietary assessment would be greatly complimented by assessment of the composition, frequency and biomass of forage available within both habitats. Such information about forage availability would assist in determining whether and how it influences diet choice in NHW. A greater understanding of what factors influence the diet of the NHW is essential for effective management of the diet, for example increasing native grasses and reducing invasive grasses. In addition, assessment of the forage community would provide insight into the spread of buffel grass throughout EFNP and RUNR, necessary information for managing and protecting the vegetation communities within NHW habitat.

Our study has further confirmed the NHW is a generalist herbivore and increased the number of known preferred dietary items. This assessment of the diet of the NHW is the first since the establishment of the translocated population at RUNR. The diet of the NHW differs significantly between the populations at EFNP and RUNR and varies significantly between seasons at each site. In comparison with the diet of the NHW reported in past studies, the contributions of specific plant taxa have changed over time, including buffel grass, which has dramatically increased in frequency and abundance in the diet. Overall, buffel grass dominated the diet, with a total abundance almost 10 times greater than any other taxon. Our study has provided an updated assessment of the diet of the critically endangered NHW at EFNP and a new assessment at RUNR that will assist with species conservation.

## AUTHOR CONTRIBUTIONS

**Fiona Casey:** Data curation (lead); formal analysis (lead); writing – original draft (lead). **Julie M. Old:** Conceptualization (equal); funding acquisition (equal); methodology (equal); project administration (equal); supervision (equal); writing – review and editing (equal). **Hayley Jade Stannard:** Conceptualization (equal); funding acquisition (equal); methodology (equal); project administration (equal); supervision (equal); writing – review and editing (equal).

## CONFLICT OF INTEREST STATEMENT

The authors declare no conflicts of interest.

## FUNDING INFORMATION

The Wombat Foundation funded the study.

## Data Availability

Data supporting this study can be found in the appendix.

## APPENDIX A

**TABLE A1:** Location of scat samples collected at Epping Forest National Park (EFNP) and Richard Underwood Nature Refuge (RUNR).

Site	Season	Collection date	Burrow ID	Latitude	Longitude
EFNP	Winter 2020	3/06/2020	B250	−22.38343	146.70387
EFNP	Winter 2020	3/06/2020	FS1	−22.38049	146.70024
EFNP	Winter 2020	3/06/2020	B275	−22.38369	146.70396
EFNP	Winter 2020	3/06/2020	B61	−22.38015	146.69836
EFNP	Winter 2020	3/06/2020	B104	−22.38062	146.69913
EFNP	Winter 2020	3/06/2020	B66	−22.37132	146.69357
EFNP	Winter 2020	3/06/2020	B48	−22.36932	146.68835
EFNP	Winter 2020	3/06/2020	FS3	−22.37201	146.6936
EFNP	Winter 2020	3/06/2020	B128	−22.37072	146.68814
EFNP	Spring 2020	3/11/2020	B273	−22.38394	146.70107
EFNP	Spring 2020	3/11/2020	B270	−22.38346	146.70074
EFNP	Spring 2020	3/11/2020	B61	−22.38023	146.69838
EFNP	Spring 2020	3/11/2020	B99	−22.37824	146.69666
EFNP	Spring 2020	3/11/2020	B62	−22.37974	146.69872
EFNP	Spring 2020	3/11/2020	B142	−22.37877	146.701
EFNP	Spring 2020	3/11/2020	B96	−22.3742	146.69402
EFNP	Spring 2020	3/11/2020	B96	−22.37413	146.69417
EFNP	Spring 2020	3/11/2020	B140	−22.37368	146.69414
EFNP	Spring 2020	3/11/2020	Adj. to B140	−22.37354	146.69427
EFNP	Spring 2020	3/11/2020	B94	−22.37314	146.69429
EFNP	Spring 2020	3/11/2020	B35	−22.36	146.69655
EFNP	Summer 2020/21	25/02/2021	B97	−22.37395	146.69327
EFNP	Summer 2020/21	25/02/2021	B71	−22.37029	146.69008
EFNP	Summer 2020/21	25/02/2021	B48	−22.36941	146.68842
EFNP	Summer 2020/21	25/02/2021	B70a	−22.36730	146.69350
EFNP	Summer 2020/21	25/02/2021	B53	−22.36957	146.68916
EFNP	Summer 2020/21	25/02/2021	B65	−22.37007	146.69269
EFNP	Summer 2020/21	25/02/2021	B96	−22.37392	146.69431
EFNP	Summer 2020/21	25/02/2021	B70	−22.36727	146.69347
EFNP	Summer 2020/21	25/02/2021	B68	−22.36862	146.69228
EFNP	Winter 2021	28/08/2021	B97	−22.37406	146.69330
EFNP	Winter 2021	28/08/2021	B73	−22.37907	146.69746
EFNP	Winter 2021	28/08/2021	B95	−22.37353	146.69353
EFNP	Winter 2021	28/08/2021	B144	−22.37291	146.69337
EFNP	Winter 2021	28/08/2021	B64	−22.37869	146.69678
EFNP	Winter 2021	28/08/2021	B96	−22.37402	146.69424
EFNP	Winter 2021	28/08/2021	B96	−22.37406	146.69414
EFNP	Winter 2021	28/08/2021	B73	−22.37907	146.69746
EFNP	Winter 2021	28/08/2021	B94	−22.37325	146.69432
EFNP	Winter 2021	28/08/2021	B99	−22.37823	146.69172
EFNP	Winter 2021	28/08/2021	B58	−22.37823	146.69172
RUNR	Winter 2020	28/06/2020	B38	−27.668572	148.706115
RUNR	Winter 2020	27/06/2020	B13	−27.670641	148.707911
RUNR	Winter 2020	28/06/2020	NA	−27.665315	148.70861
RUNR	Winter 2020	27/06/2020	B36	−27.667623	148.704716
RUNR	Winter 2020	28/06/2020	WD46	−27.66369	148.70854
RUNR	Winter 2020	28/06/2020	B40	−27.66416	148.702688
RUNR	Winter 2020	27/06/2020	B31	−27.663418	148.705949
RUNR	Winter 2020	28/06/2020	B3	−27.666153	148.704067
RUNR	Spring 2020	27/09/2020	B31	−27.663418	148.705949
RUNR	Spring 2020	27/09/2020	NA	−27.662790	148.70605
RUNR	Spring 2020	27/09/2020	B3	−27.666153	148.704067
RUNR	Spring 2020	27/09/2020	B30	−27.665247	148.70401
RUNR	Spring 2020	28/09/2020	B41	−27.668821	148.707124
RUNR	Spring 2020	27/09/2020	B4	−27.665560	148.70506
RUNR	Spring 2020	28/09/2020	B29	−27.669650	148.70554
RUNR	Spring 2020	28/09/2020	B27	−27.669834	148.711217
RUNR	Summer 2020/21	5/01/2021	B43	−27.665960	148.707921
RUNR	Summer 2020/21	5/01/2021	B41	−27.668821	148.707120
RUNR	Summer 2020/21	6/01/2021	B40	−27.66416	148.702688
RUNR	Summer 2020/21	6/01/2021	WD19	−27.669609	148.706216
RUNR	Summer 2020/21	6/01/2021	B29	−27.669796	148.705573
RUNR	Summer 2020/21	6/01/2021	B31	−27.663418	148.705949
RUNR	Summer 2020/21	6/01/2021	B22	−27.662450	148.701432
RUNR	Summer 2020/21	6/01/2021	B38	−27.668572	148.706115
RUNR	Autumn 2021	14/04/2021	B29	−27.669796	148.705573
RUNR	Autumn 2021	15/04/2021	B77	−22.663690	148.708540
RUNR	Autumn 2021	14/04/2021	B8	−22.666253	148.708256
RUNR	Autumn 2021	14/04/2021	NA	−22.666830	148.702930
RUNR	Autumn 2021	14/04/2021	B33	−22.669379	148.705230
RUNR	Autumn 2021	14/04/2021	B33	−22.666153	148.704067
RUNR	Autumn 2021	14/04/2021	B17	−22.669870	148.702844
RUNR	Autumn 2021	15/04/2021	B27	−22.669834	148.711217
RUNR	Winter 2021	7/07/2021	B8	−27.66253	148.708256
RUNR	Winter 2021	7/07/2021	B31	−27.663418	148.705949
RUNR	Winter 2021	7/07/2021	B49	−27.663479	148.698654
RUNR	Winter 2021	7/07/2021	B28	−27.671855	148.710305
RUNR	Winter 2021	7/07/2021	B22	−27.66245	148.701432
RUNR	Winter 2021	8/07/2021	B38	−27.668572	148.706115
RUNR	Winter 2021	8/07/2021	B13	−27.670641	148.707911
RUNR	Winter 2021	8/07/2021	B12	−27.670108	148.707762
RUNR	Spring 2021	2/09/2021	B31	−27.66341	148.705945
RUNR	Spring 2021	2/09/2021	B37	−27.66831	148.70379
RUNR	Spring 2021	2/09/2021	B8	−27.66625	148.70825
RUNR	Spring 2021	2/09/2021	B6	−27.66445	148.70674
RUNR	Spring 2021	2/09/2021	B29	−27.66979	148.70557
RUNR	Spring 2021	2/09/2021	B30	−27.66524	148.70471
RUNR	Spring 2021	2/09/2021	B15	−27.66679	148.70855
RUNR	Spring 2021	2/09/2021	NA	−27.66684	148.70293

**TABLE A2:** Number of scat samples from Epping Forest National Park (EFNP) in which each taxon was present (frequency of occurrence) during each sampling period from 2020 to 2021.

Taxon							Season				
Kingdom	Phylum	Class	Order	Family	Genus	Species	2020/21 Total	Winter 2020	Spring 2020	Summer 2020/21	Winter 2021
Number of samples per sampling period							36	7	12	9	8
Unassigned							36	7	12	9	8
Fungi							1	0	1	0	0
Fungi	Ascomycota	Dothideomycetes	Pleosporales	Didymellaceae	Didymella		*1*	0	0	0	1
Fungi	Ascomycota	Dothideomycetes	Pleosporales	Didymellaceae	Epicoccum	nigrum	*1*	0	1	0	0
Fungi	Ascomycota	Dothideomycetes	Pleosporales	Sporormiaceae	Preussia		*4*	0	0	0	4
Fungi	Ascomycota	Sordariomycetes	Hypocreales	Hypocreaceae	Trichoderma		*1*	0	0	0	1
Fungi	Ascomycota	Sordariomycetes	Hypocreales	Incertae sedis	Acremonium	persicinum	*0*	0	0	0	0
Fungi	Basidiomycota	Cystobasidiomycetes	Cystobasidiales	Cystobasidiaceae			1	0	0	0	1
Fungi	Basidiomycota	Cystobasidiomycetes	Cystobasidiales	Cystobasidiaceae	Cystobasidium	benthicum	5	0	1	0	4
Fungi	Basidiomycota	Tremellomycetes	Filobasidiales	Filobasidiaceae	Naganishia	albida	1	0	0	0	1
Fungi	Basidiomycota	Tremellomycetes	Filobasidiales	Piskurozymaceae	Solicoccozyma	phenolica	1	0	0	0	1
Viridiplantae							1	0	0	0	1
Viridiplantae	Anthophyta	Eudicotyledonae					4	1	0	1	2
Viridiplantae	Anthophyta	Eudicotyledonae	Asterales	Asteraceae			2	1	1	0	0
Viridiplantae	Anthophyta	Eudicotyledonae	Asterales	Asteraceae	Pterocaulon	sphacelatum^a^	2	0	0	0	2
Viridiplantae	Anthophyta	Eudicotyledonae	Asterales	Asteraceae	*Taraxacum*		1	0	1	0	0
Viridiplantae	Anthophyta	Eudicotyledonae	Asterales	Asteraceae	Vittadinia	dissecta^a^	1	0	1	0	0
Viridiplantae	Anthophyta	Eudicotyledonae	Brassicales	Cleomaceae	Cleome	cleomoides^a^	4	0	0	4	0
Viridiplantae	Anthophyta	Eudicotyledonae	Caryophyllales	Aizoaceae	Trianthema		1	0	0	0	1
Viridiplantae	Anthophyta	Eudicotyledonae	Caryophyllales	Amaranthaceae	Alternanthera	sessilis	1	0	0	1	0
Viridiplantae	Anthophyta	Eudicotyledonae	Caryophyllales	Amaranthaceae	Salsola		1	0	0	0	1
Viridiplantae	Anthophyta	Eudicotyledonae	Caryophyllales	Molluginaceae	Hypertelis		1	0	0	1	0
Viridiplantae	Anthophyta	Eudicotyledonae	Caryophyllales	Nyctaginaceae	Boerhavia	erecta	20	6	4	9	1
Viridiplantae	Anthophyta	Eudicotyledonae	Caryophyllales	Portulacaceae	Portulaca		4	0	0	3	1
Viridiplantae	Anthophyta	Eudicotyledonae	Caryophyllales	Portulacaceae	Portulaca	oleracea	2	0	0	1	1
Viridiplantae	Anthophyta	Eudicotyledonae	Celastrales	Celastraceae	Denhamia	celastroides^a^	1	0	0	1	0
Viridiplantae	Anthophyta	Eudicotyledonae	Cucurbitales	Cucurbitaceae	Cucumis	sativus	1	0	0	0	1
Viridiplantae	Anthophyta	Eudicotyledonae	Fabales	Fabaceae			3	0	3	0	0
Viridiplantae	Anthophyta	Eudicotyledonae	Fabales	Fabaceae	Acacia		7	1	1	3	2
Viridiplantae	Anthophyta	Eudicotyledonae	Fabales	Fabaceae	Acacia	ampliceps^a^	4	1	3	0	0
Viridiplantae	Anthophyta	Eudicotyledonae	Fabales	Fabaceae	Acacia	confusa	1	1	0	0	0
Viridiplantae	Anthophyta	Eudicotyledonae	Fabales	Fabaceae	Bauhinia	apertilobata^a^	6	2	3	1	0
Viridiplantae	Anthophyta	Eudicotyledonae	Fabales	Fabaceae	Chamaecrista		2	1	0	0	1
Viridiplantae	Anthophyta	Eudicotyledonae	Fabales	Fabaceae	Desmodium		4	0	0	3	1
Viridiplantae	Anthophyta	Eudicotyledonae	Fabales	Fabaceae	Desmodium	triflorum	1	0	0	1	0
Viridiplantae	Anthophyta	Eudicotyledonae	Fabales	Fabaceae	Galactia	striata	4	0	0	2	2
Viridiplantae	Anthophyta	Eudicotyledonae	Fabales	Fabaceae	Glycine	clandestina^a^	0	0	0	0	0
Viridiplantae	Anthophyta	Eudicotyledonae	Fabales	Fabaceae	Indigofera	astragalina^a^	1	0	0	0	1
Viridiplantae	Anthophyta	Eudicotyledonae	Fabales	Fabaceae	Indigofera	colutea	10	1	0	5	4
Viridiplantae	Anthophyta	Eudicotyledonae	Fabales	Fabaceae	Indigofera	linifolia^a^	4	0	0	4	0
Viridiplantae	Anthophyta	Eudicotyledonae	Fabales	Fabaceae	Indigofera	linnaei^a^	1	0	0	1	0
Viridiplantae	Anthophyta	Eudicotyledonae	Fabales	Fabaceae	Neptunia	monosperma^a^	1	1	0	0	0
Viridiplantae	Anthophyta	Eudicotyledonae	Fabales	Fabaceae	Rhynchosia	minima^a^	1	0	0	0	1
Viridiplantae	Anthophyta	Eudicotyledonae	Fabales	Fabaceae	Tephrosia		1	0	0	1	0
Viridiplantae	Anthophyta	Eudicotyledonae	Fabales	Fabaceae	Zornia	gibbosa	5	0	0	5	0
Viridiplantae	Anthophyta	Eudicotyledonae	Fagales	Juglandaceae	Juglans	nigra	1	0	1	0	0
Viridiplantae	Anthophyta	Eudicotyledonae	Gentianales	Apocynaceae			1	0	0	1	0
Viridiplantae	Anthophyta	Eudicotyledonae	Gentianales	Apocynaceae	Alstonia	scholaris^a^	1	0	0	0	1
Viridiplantae	Anthophyta	Eudicotyledonae	Lamiales	Acanthaceae	Justicia	procumbens	1	0	0	1	0
Viridiplantae	Anthophyta	Eudicotyledonae	Lamiales	Acanthaceae	Ruellia	patula	1	0	0	0	1
Viridiplantae	Anthophyta	Eudicotyledonae	Lamiales	Lamiaceae			1	1	0	0	0
Viridiplantae	Anthophyta	Eudicotyledonae	Lamiales	Oleaceae	Jasminum	fluminense	0	0	0	0	0
Viridiplantae	Anthophyta	Eudicotyledonae	Malpighiales	Euphorbiaceae	Euphorbia	humifusa	3	2	0	1	0
Viridiplantae	Anthophyta	Eudicotyledonae	Malpighiales	Phyllanthaceae	Phyllanthus	ussuriensis	4	1	0	1	2
Viridiplantae	Anthophyta	Eudicotyledonae	Malpighiales	Phyllanthaceae	Synostemon		3	0	1	1	1
Viridiplantae	Anthophyta	Eudicotyledonae	Malvales	Malvaceae			13	2	2	3	6
Viridiplantae	Anthophyta	Eudicotyledonae	Malvales	Malvaceae	Abutilon	indicum^a^	1	0	1	0	0
Viridiplantae	Anthophyta	Eudicotyledonae	Malvales	Malvaceae	Hibiscus		6	1	3	1	1
Viridiplantae	Anthophyta	Eudicotyledonae	Malvales	Malvaceae	Hibiscus	micranthus	1	1	0	0	0
Viridiplantae	Anthophyta	Eudicotyledonae	Malvales	Malvaceae	Malvastrum	coromandelianum	5	1	0	0	4
Viridiplantae	Anthophyta	Eudicotyledonae	Malvales	Malvaceae	Trochetiopsis	erythroxylon	18	5	7	5	1
Viridiplantae	Anthophyta	Eudicotyledonae	Malvales	Malvaceae	Waltheria	indica	20	6	2	5	7
Viridiplantae	Anthophyta	Eudicotyledonae	Myrtales	Myrtaceae			2	0	2	0	0
Viridiplantae	Anthophyta	Eudicotyledonae	Myrtales	Myrtaceae	Angophora	bakeri^a^	5	1	1	3	0
Viridiplantae	Anthophyta	Eudicotyledonae	Myrtales	Myrtaceae	Corymbia	ficifolia^a^	9	0	3	6	0
Viridiplantae	Anthophyta	Eudicotyledonae	Myrtales	Myrtaceae	Eucalyptus		5	0	3	2	0
Viridiplantae	Anthophyta	Eudicotyledonae	Myrtales	Myrtaceae	Eucalyptus	brachyandra^a^	9	2	5	2	0
Viridiplantae	Anthophyta	Eudicotyledonae	Myrtales	Myrtaceae	Melaleuca	armillaris^a^	1	1	0	0	0
Viridiplantae	Anthophyta	Eudicotyledonae	Myrtales	Myrtaceae	Melaleuca	quinquenervia^a^	1	0	0	0	1
Viridiplantae	Anthophyta	Eudicotyledonae	Oxalidales	Oxalidaceae	Oxalis	corniculata	1	0	0	0	1
Viridiplantae	Anthophyta	Eudicotyledonae	Rosales	Rhamnaceae	Ventilago	denticulata	2	0	0	2	0
Viridiplantae	Anthophyta	Eudicotyledonae	Sapindales	Meliaceae			1	0	0	1	0
Viridiplantae	Anthophyta	Eudicotyledonae	Sapindales	Rutaceae	Geijera	parviflora^a^	2	0	2	0	0
Viridiplantae	Anthophyta	Eudicotyledonae	Sapindales	Rutaceae			1	0	0	0	1
Viridiplantae	Anthophyta	Eudicotyledonae	Sapindales	Sapindaceae	Alectryon	excelsus	2	0	2	0	0
Viridiplantae	Anthophyta	Eudicotyledonae	Solanales	Convolvulaceae	Evolvulus	alsinoides^a^	24	4	7	8	5
Viridiplantae	Anthophyta	Eudicotyledonae	Solanales	Convolvulaceae	Ipomoea		1	0	0	1	0
Viridiplantae	Anthophyta	Eudicotyledonae	Solanales	Convolvulaceae	Ipomoea	oenotherae	1	0	0	1	0
Viridiplantae	Anthophyta	Eudicotyledonae	Solanales	Convolvulaceae	Ipomoea	polymorpha^a^	3	0	0	3	0
Viridiplantae	Anthophyta	Eudicotyledonae	Solanales	Solanaceae	Solanum	dianthophorum^a^	2	1	1	0	0
Viridiplantae	Anthophyta	Eudicotyledonae	Solanales	Solanaceae			1	1	0	0	0
Viridiplantae	Anthophyta	Eudicotyledonae	Vitales	Vitaceae	Vitis	heyneana	5	1	2	0	2
Viridiplantae	Anthophyta	Eudicotyledonae	Zygophyllales	Zygophyllaceae	Tribulus		10	0	2	8	0
Viridiplantae	Anthophyta	Eudicotyledonae	Zygophyllales	Zygophyllaceae	Tribulus	pentandrus	6	0	0	6	0
Viridiplantae	Anthophyta	Eudicotyledonae	Zygophyllales	Zygophyllaceae	Tribulus	terrestris	1	0	0	1	0
Viridiplantae	Anthophyta	Monocotyledonae	Poales	Cyperaceae	Bulbostylis	barbata^a^	5	0	0	5	0
Viridiplantae	Anthophyta	Monocotyledonae	Poales	Cyperaceae	Cyperus		1	0	0	1	0
Viridiplantae	Anthophyta	Monocotyledonae	Poales	Cyperaceae	Fimbristylis		35	7	12	9	7
Viridiplantae	Anthophyta	Monocotyledonae	Poales	Cyperaceae	Fimbristylis	dichotoma^a^	4	0	1	2	1
Viridiplantae	Anthophyta	Monocotyledonae	Poales	Cyperaceae	Fimbristylis	stauntonii	1	0	0	1	0
Viridiplantae	Anthophyta	Monocotyledonae	Poales	Poaceae			22	2	8	9	3
Viridiplantae	Anthophyta	Monocotyledonae	Poales	Poaceae	Acrachne	racemosa^a^	1	0	0	1	0
Viridiplantae	Anthophyta	Monocotyledonae	Poales	Poaceae	Aristida		12	1	4	4	3
Viridiplantae	Anthophyta	Monocotyledonae	Poales	Poaceae	Aristida	calycina^a^	11	1	5	4	1
Viridiplantae	Anthophyta	Monocotyledonae	Poales	Poaceae	Aristida	caput‐medusae^a^	15	1	1	9	4
Viridiplantae	Anthophyta	Monocotyledonae	Poales	Poaceae	Aristida	holathera^a^	2	0	0	2	0
Viridiplantae	Anthophyta	Monocotyledonae	Poales	Poaceae	Aristida	ingrata^a^	13	1	1	8	3
Viridiplantae	Anthophyta	Monocotyledonae	Poales	Poaceae	Arundinella	bengalensis	11	1	6	3	1
Viridiplantae	Anthophyta	Monocotyledonae	Poales	Poaceae	Avena	ventricosa	1	0	0	0	1
Viridiplantae	Anthophyta	Monocotyledonae	Poales	Poaceae	Bothriochloa		6	4	1	1	0
Viridiplantae	Anthophyta	Monocotyledonae	Poales	Poaceae	Bothriochloa	pertusa	8	3	2	2	1
Viridiplantae	Anthophyta	Monocotyledonae	Poales	Poaceae	Brachiaria		2	1	0	1	0
Viridiplantae	Anthophyta	Monocotyledonae	Poales	Poaceae	Brachiaria	ramosa	1	1	0	0	0
Viridiplantae	Anthophyta	Monocotyledonae	Poales	Poaceae	Brachiaria	subquadripara	14	3	2	9	0
Viridiplantae	Anthophyta	Monocotyledonae	Poales	Poaceae	Cenchrus	ciliaris	36	7	12	9	8
Viridiplantae	Anthophyta	Monocotyledonae	Poales	Poaceae	Chloris	pumilio^a^	1	0	0	1	0
Viridiplantae	Anthophyta	Monocotyledonae	Poales	Poaceae	Chrysopogon		19	4	8	6	1
Viridiplantae	Anthophyta	Monocotyledonae	Poales	Poaceae	Chrysopogon	latifolius^a^	35	7	12	9	7
Viridiplantae	Anthophyta	Monocotyledonae	Poales	Poaceae	Dactyloctenium	aegyptium	8	0	0	8	0
Viridiplantae	Anthophyta	Monocotyledonae	Poales	Poaceae	Dinebra		2	0	0	2	0
Viridiplantae	Anthophyta	Monocotyledonae	Poales	Poaceae	Enneapogon	scoparius	27	4	8	9	6
Viridiplantae	Anthophyta	Monocotyledonae	Poales	Poaceae	Enteropogon	ramosus^a^	3	1	0	2	0
Viridiplantae	Anthophyta	Monocotyledonae	Poales	Poaceae	Eragrostis		2	0	1	1	0
Viridiplantae	Anthophyta	Monocotyledonae	Poales	Poaceae	Eragrostis	aegyptiaca	1	0	0	1	0
Viridiplantae	Anthophyta	Monocotyledonae	Poales	Poaceae	Eragrostis	andicola	1	0	0	1	0
Viridiplantae	Anthophyta	Monocotyledonae	Poales	Poaceae	Eragrostis	ciliaris	1	0	0	1	0
Viridiplantae	Anthophyta	Monocotyledonae	Poales	Poaceae	Eragrostis	confertiflora^a^	2	0	0	2	0
Viridiplantae	Anthophyta	Monocotyledonae	Poales	Poaceae	Eragrostis	refracta	9	0	0	9	0
Viridiplantae	Anthophyta	Monocotyledonae	Poales	Poaceae	Eriachne		1	0	0	1	0
Viridiplantae	Anthophyta	Monocotyledonae	Poales	Poaceae	Eulalia	aurea^a^	4	0	3	1	0
Viridiplantae	Anthophyta	Monocotyledonae	Poales	Poaceae	Heteropogon	contortus^a^	13	4	2	3	4
Viridiplantae	Anthophyta	Monocotyledonae	Poales	Poaceae	Melinis	repens	1	0	0	0	1
Viridiplantae	Anthophyta	Monocotyledonae	Poales	Poaceae	Panicum		0	0	0	0	0
Viridiplantae	Anthophyta	Monocotyledonae	Poales	Poaceae	Perotis	hordeiformis	13	1	1	9	2
Viridiplantae	Anthophyta	Monocotyledonae	Poales	Poaceae	Setaria	viridis	1	0	0	1	0
Viridiplantae	Anthophyta	Monocotyledonae	Poales	Poaceae	Sporobolus	carolii^a^	2	0	0	2	0
Viridiplantae	Anthophyta	Monocotyledonae	Poales	Poaceae	Themeda	triandra^a^	3	0	0	2	1
Viridiplantae	Anthophyta	Monocotyledonae	Poales	Poaceae	Themeda	villosa	0	0	0	0	0
Viridiplantae	Anthophyta	Monocotyledonae	Poales	Poaceae	Thyridolepis	mitchelliana^a^	1	0	0	0	1
Viridiplantae	Anthophyta	Monocotyledonae	Poales	Poaceae	Tripogon	loliiformis^a^	7	2	4	1	0
Viridiplantae	Anthophyta	Monocotyledonae	Poales	Poaceae	Willkommia	texana	4	0	0	4	0
Viridiplantae	Anthophyta	Monocotyledonae	Zingiberales	Musaceae	Musa	mannii^a^	0	0	0	0	0
Viridiplantae	Coniferophyta	Pinopsida	Pinales	Pinaceae	Pinus	hartwegii	3	0	1	1	1

^a^ Native species.

**TABLE A3:** Number of scat samples from Richard Underwood Nature Refuge (RUNR) in which each taxon was present (frequency of occurrence) during each sampling period from 2020 to 2021.

Taxon							Season					
Kingdom	Phylum	Class	Order	Family	Genus	Species	Total	Winter 2020	Summer 2020/21	Autumn 2021	Winter 2021	Spring 2021
Number of samples per sampling period							38	6	8	8	8	8
Unassigned							33	6	6	5	8	8
Fungi							1	0	1	0	0	0
Fungi	Ascomycota	Dothideomycetes	Capnodiales	Cladosporiaceae	*Cladosporium*		1	0	0	1	0	0
Fungi	Ascomycota	Dothideomycetes	Pleosporales	Didymellaceae	*Epicoccum*		1	1	0	0	0	0
Fungi	Ascomycota	Dothideomycetes	Pleosporales	Sporormiaceae	*Preussia*		5	0	5	0	0	0
Fungi	Ascomycota	Leotiomycetes	Helotiales	Helotiaceae	*Acidea*	*extrema*	1	0	0	1	0	0
Fungi	Ascomycota	Leotiomycetes	Helotiales	Hyaloscyphaceae			1	0	0	0	1	0
Fungi	Ascomycota	Sordariomycetes	Hypocreales	Hypocreaceae	*Trichoderma*		1	0	0	0	0	1
Fungi	Basidiomycota	Agaricomycetes					3	0	2	0	0	1
Fungi	Basidiomycota	Agaricomycetes	Phallales	Clathraceae	*Ileodictyon*	*gracile*	1	0	0	0	0	1
Fungi	Basidiomycota	Agaricomycetes	Polyporales	Phanerochaetaceae	*Oxychaete*		1	0	0	0	1	0
Fungi	Basidiomycota	Agaricomycetes	Russulales	Peniophoraceae	*Peniophora*		2	0	0	0	2	0
Fungi	Basidiomycota	Cystobasidiomycetes	Cystobasidiales	Cystobasidiaceae			1	0	0	0	0	1
Fungi	Basidiomycota	Cystobasidiomycetes	Cystobasidiales	Cystobasidiaceae	*Cystobasidium*	*benthicum*	2	0	2	0	0	0
Fungi	Basidiomycota	Tremellomycetes	Filobasidiales	Filobasidiaceae	*Naganishia*	*albida*	2	0	2	0	0	0
Viridiplantae	Anthophyta	Eudicotyledonae					6	2	0	1	1	2
Viridiplantae	Anthophyta	Eudicotyledonae	Asterales	Asteraceae			8	2	1	3	0	2
Viridiplantae	Anthophyta	Eudicotyledonae	Asterales	Asteraceae	*Calotis*		12	3	2	5	0	2
Viridiplantae	Anthophyta	Eudicotyledonae	Asterales	Asteraceae	*Lactuca*	*virosa* ^a^	1	0	0	0	0	1
Viridiplantae	Anthophyta	Eudicotyledonae	Asterales	Asteraceae	*Podolepis*		1	0	0	0	0	1
Viridiplantae	Anthophyta	Eudicotyledonae	Asterales	Asteraceae	*Podolepis*	*robusta* ^a^	1	0	0	0	0	1
Viridiplantae	Anthophyta	Eudicotyledonae	Asterales	Asteraceae	*Pterocaulon*	*sphacelatum* ^a^	2	2	0	0	0	0
Viridiplantae	Anthophyta	Eudicotyledonae	Asterales	Asteraceae	*Vittadinia*	*dissecta* ^a^	2	1	0	1	0	0
Viridiplantae	Anthophyta	Eudicotyledonae	Brassicales	Brassicaceae			3	2	0	0	1	0
Viridiplantae	Anthophyta	Eudicotyledonae	Brassicales	Brassicaceae	*Brassica*	*carinata*	3	0	0	0	2	1
Viridiplantae	Anthophyta	Eudicotyledonae	Caryophyllales	Amaranthaceae			1	0	0	0	0	1
Viridiplantae	Anthophyta	Eudicotyledonae	Caryophyllales	Amaranthaceae	*Alternanthera*		3	3	0	0	0	0
Viridiplantae	Anthophyta	Eudicotyledonae	Caryophyllales	Amaranthaceae	*Alternanthera*	*sessilis*	11	4	2	5	0	0
Viridiplantae	Anthophyta	Eudicotyledonae	Caryophyllales	Amaranthaceae	*Chenopodium*		19	3	2	5	3	6
Viridiplantae	Anthophyta	Eudicotyledonae	Caryophyllales	Amaranthaceae	*Chenopodium*	*opulifolium*	1	0	0	0	0	1
Viridiplantae	Anthophyta	Eudicotyledonae	Caryophyllales	Amaranthaceae	*Dysphania*		10	5	0	3	0	2
Viridiplantae	Anthophyta	Eudicotyledonae	Caryophyllales	Amaranthaceae	*Ptilotus*	*polystachyus* ^a^	4	3	0	1	0	0
Viridiplantae	Anthophyta	Eudicotyledonae	Caryophyllales	Nyctaginaceae	*Boerhavia*	*erecta*	27	6	8	8	2	3
Viridiplantae	Anthophyta	Eudicotyledonae	Caryophyllales	Portulacaceae	*Portulaca*		2	1	0	1	0	0
Viridiplantae	Anthophyta	Eudicotyledonae	Caryophyllales	Portulacaceae	*Portulaca*	*oleracea*	1	1	0	0	0	0
Viridiplantae	Anthophyta	Eudicotyledonae	Cucurbitales	Cucurbitaceae	*Cucumis*	*sativus*	4	0	1	0	2	1
Viridiplantae	Anthophyta	Eudicotyledonae	Ericales	Theaceae	*Camellia*		1	0	0	0	1	0
Viridiplantae	Anthophyta	Eudicotyledonae	Fabales	Fabaceae	*Acacia*	*confusa*	18	3	4	3	4	4
Viridiplantae	Anthophyta	Eudicotyledonae	Fabales	Fabaceae	*Glycine*	*clandestina* ^a^	13	3	2	5	2	1
Viridiplantae	Anthophyta	Eudicotyledonae	Fabales	Fabaceae	*Medicago*	*minima*	1	0	0	0	0	1
Viridiplantae	Anthophyta	Eudicotyledonae	Gentianales	Apocynaceae	*Parsonsia*	*crebriflora* ^a^	1	0	0	0	0	1
Viridiplantae	Anthophyta	Eudicotyledonae	Geraniales	Geraniaceae	*Erodium*		2	0	0	0	0	2
Viridiplantae	Anthophyta	Eudicotyledonae	Lamiales				13	3	5	3	0	2
Viridiplantae	Anthophyta	Eudicotyledonae	Lamiales	Acanthaceae	*Justicia*	*procumbens*	2	0	0	2	0	0
Viridiplantae	Anthophyta	Eudicotyledonae	Lamiales	Lamiaceae			5	2	1	0	0	2
Viridiplantae	Anthophyta	Eudicotyledonae	Lamiales	Oleaceae	*Fraxinus*	*velutina*	1	0	1	0	0	0
Viridiplantae	Anthophyta	Eudicotyledonae	Lamiales	Oleaceae	*Jasminum*	*fluminense*	5	0	1	3	1	0
Viridiplantae	Anthophyta	Eudicotyledonae	Lamiales	Scrophulariaceae	*Myoporum*	*insulare* ^a^	1	0	1	0	0	0
Viridiplantae	Anthophyta	Eudicotyledonae	Malpighiales	Euphorbiaceae	*Euphorbia*	*humifusa*	2	0	0	1	0	1
Viridiplantae	Anthophyta	Eudicotyledonae	Malpighiales	Phyllanthaceae	*Phyllanthus*	*ussuriensis*	4	0	0	2	0	2
Viridiplantae	Anthophyta	Eudicotyledonae	Malpighiales	Phyllanthaceae	*Synostemon*		1	0	0	1	0	0
Viridiplantae	Anthophyta	Eudicotyledonae	Malvales	Malvaceae			23	4	4	7	4	4
Viridiplantae	Anthophyta	Eudicotyledonae	Malvales	Malvaceae	*Abutilon*	*indicum* ^a^	18	4	3	7	1	3
Viridiplantae	Anthophyta	Eudicotyledonae	Malvales	Malvaceae	*Hibiscus*		21	3	2	7	3	6
Viridiplantae	Anthophyta	Eudicotyledonae	Malvales	Malvaceae	*Hibiscus*	*micranthus*	5	1	1	1	1	1
Viridiplantae	Anthophyta	Eudicotyledonae	Malvales	Malvaceae	*Malvastrum*	*coromandelianum*	25	4	2	6	6	7
Viridiplantae	Anthophyta	Eudicotyledonae	Malvales	Malvaceae	*Trochetiopsis*	*erythroxylon*	8	0	3	1	0	4
Viridiplantae	Anthophyta	Eudicotyledonae	Myrtales	Myrtaceae	*Angophora*	*bakeri* ^a^	5	2	1	2	0	0
Viridiplantae	Anthophyta	Eudicotyledonae	Myrtales	Myrtaceae	*Angophora*	*costata* ^a^	1	0	1	0	0	0
Viridiplantae	Anthophyta	Eudicotyledonae	Myrtales	Myrtaceae	*Corymbia*	*ficifolia* ^a^	4	0	4	0	0	0
Viridiplantae	Anthophyta	Eudicotyledonae	Myrtales	Myrtaceae	*Eucalyptus*		7	2	3	1	1	0
Viridiplantae	Anthophyta	Eudicotyledonae	Myrtales	Myrtaceae	*Eucalyptus*	*brachyandra* ^a^	9	5	3	0	0	1
Viridiplantae	Anthophyta	Eudicotyledonae	Oxalidales	Oxalidaceae	*Oxalis*	*corniculata*	9	0	0	1	2	6
Viridiplantae	Anthophyta	Eudicotyledonae	Proteales	Proteaceae	*Grevillea*	*robusta* ^a^	1	0	0	0	1	0
Viridiplantae	Anthophyta	Eudicotyledonae	Rosales	Urticaceae			1	0	1	0	0	0
Viridiplantae	Anthophyta	Eudicotyledonae	Rosales	Urticaceae	*Urtica*		1	0	1	0	0	0
Viridiplantae	Anthophyta	Eudicotyledonae	Sapindales	Rutaceae	*Citrus*	*reticulata*	1	0	0	0	1	0
Viridiplantae	Anthophyta	Eudicotyledonae	Sapindales	Rutaceae	*Geijera*	*parviflora* ^a^	18	3	3	4	4	4
Viridiplantae	Anthophyta	Eudicotyledonae	Sapindales	Sapindaceae	*Alectryon*	*excelsus*	5	1	3	0	0	1
Viridiplantae	Anthophyta	Eudicotyledonae	Saxifragales	Crassulaceae	*Crassula*	*campestris*	8	1	0	0	0	7
Viridiplantae	Anthophyta	Eudicotyledonae	Saxifragales	Haloragaceae	*Haloragis*	*erecta*	1	0	0	1	0	0
Viridiplantae	Anthophyta	Eudicotyledonae	Solanales	Convolvulaceae	*Evolvulus*	*alsinoides* ^a^	31	4	7	8	4	8
Viridiplantae	Anthophyta	Eudicotyledonae	Solanales	Solanaceae	*Capsicum*		1	0	0	0	1	0
Viridiplantae	Anthophyta	Eudicotyledonae	Solanales	Solanaceae	*Solanum*		12	2	0	1	3	6
Viridiplantae	Anthophyta	Eudicotyledonae	Solanales	Solanaceae	*Solanum*	*dianthophorum* ^a^	12	0	1	1	4	6
Viridiplantae	Anthophyta	Eudicotyledonae	Solanales	Solanaceae	*Solanum*	*tuberosum*	1	0	1	0	0	0
Viridiplantae	Anthophyta	Eudicotyledonae	Vitales	Vitaceae	*Vitis*	*heyneana*	3	1	0	1	0	1
Viridiplantae	Anthophyta	Monocotyledonae	Asparagales	Xanthorrhoeaceae	*Dianella*	*longifolia* ^a^	1	0	0	0	0	1
Viridiplantae	Anthophyta	Monocotyledonae	Poales	Cyperaceae	*Bulbostylis*	*barbata* ^a^	3	3	0	0	0	0
Viridiplantae	Anthophyta	Monocotyledonae	Poales	Cyperaceae	*Cyperus*		13	2	2	3	0	6
Viridiplantae	Anthophyta	Monocotyledonae	Poales	Cyperaceae	*Eleocharis*		1	0	0	1	0	0
Viridiplantae	Anthophyta	Monocotyledonae	Poales	Cyperaceae	*Fimbristylis*		4	1	2	0	0	1
Viridiplantae	Anthophyta	Monocotyledonae	Poales	Poaceae			38	6	8	8	8	8
Viridiplantae	Anthophyta	Monocotyledonae	Poales	Poaceae	*Aristida*		31	4	8	8	4	7
Viridiplantae	Anthophyta	Monocotyledonae	Poales	Poaceae	*Aristida*	*calycina* ^a^	19	3	7	6	0	3
Viridiplantae	Anthophyta	Monocotyledonae	Poales	Poaceae	*Aristida*	*caput‐medusae* ^a^	22	5	6	6	2	3
Viridiplantae	Anthophyta	Monocotyledonae	Poales	Poaceae	*Aristida*	*congesta*	1	0	0	1	0	0
Viridiplantae	Anthophyta	Monocotyledonae	Poales	Poaceae	*Austrostipa*	*nodosa* ^a^	14	3	2	5	2	2
Viridiplantae	Anthophyta	Monocotyledonae	Poales	Poaceae	*Avena*	*ventricosa*	5	0	0	1	2	2
Viridiplantae	Anthophyta	Monocotyledonae	Poales	Poaceae	*Bothriochloa*	*ischaemum*	7	2	0	4	0	1
Viridiplantae	Anthophyta	Monocotyledonae	Poales	Poaceae	*Brachiaria*		3	0	0	1	1	1
Viridiplantae	Anthophyta	Monocotyledonae	Poales	Poaceae	*Brachiaria*	*ramosa*	11	2	2	3	3	1
Viridiplantae	Anthophyta	Monocotyledonae	Poales	Poaceae	*Brachiaria*	*subquadripara*	9	6	1	2	0	0
Viridiplantae	Anthophyta	Monocotyledonae	Poales	Poaceae	*Cenchrus*		1	0	0	0	0	1
Viridiplantae	Anthophyta	Monocotyledonae	Poales	Poaceae	*Cenchrus*	*ciliaris*	38	6	8	8	8	8
Viridiplantae	Anthophyta	Monocotyledonae	Poales	Poaceae	*Chloris*		12	2	0	3	0	7
Viridiplantae	Anthophyta	Monocotyledonae	Poales	Poaceae	*Chloris*	*castilloniana*	4	0	0	2	0	2
Viridiplantae	Anthophyta	Monocotyledonae	Poales	Poaceae	*Chloris*	*pumilio* ^a^	1	0	1	0	0	0
Viridiplantae	Anthophyta	Monocotyledonae	Poales	Poaceae	*Chloris*	*ventricosa* ^a^	1	0	0	1	0	0
Viridiplantae	Anthophyta	Monocotyledonae	Poales	Poaceae	*Chrysopogon*		6	1	0	4	1	0
Viridiplantae	Anthophyta	Monocotyledonae	Poales	Poaceae	*Chrysopogon*	*latifolius* ^a^	9	2	2	5	0	0
Viridiplantae	Anthophyta	Monocotyledonae	Poales	Poaceae	*Dactyloctenium*	*aegyptium*	1	1	0	0	0	0
Viridiplantae	Anthophyta	Monocotyledonae	Poales	Poaceae	*Digitaria*		16	2	3	3	1	7
Viridiplantae	Anthophyta	Monocotyledonae	Poales	Poaceae	*Dinebra*		2	1	0	1	0	0
Viridiplantae	Anthophyta	Monocotyledonae	Poales	Poaceae	*Enneapogon*	*scoparius*	20	5	3	8	1	3
Viridiplantae	Anthophyta	Monocotyledonae	Poales	Poaceae	*Enteropogon*		1	0	0	1	0	0
Viridiplantae	Anthophyta	Monocotyledonae	Poales	Poaceae	*Enteropogon*	*ramosus* ^a^	18	4	2	6	1	5
Viridiplantae	Anthophyta	Monocotyledonae	Poales	Poaceae	*Eragrostis*		11	4	1	3	0	3
Viridiplantae	Anthophyta	Monocotyledonae	Poales	Poaceae	*Eragrostis*	*andicola*	2	2	0	0	0	0
Viridiplantae	Anthophyta	Monocotyledonae	Poales	Poaceae	*Eragrostis*	*curvula*	16	1	3	5	0	7
Viridiplantae	Anthophyta	Monocotyledonae	Poales	Poaceae	*Heteropogon*	*contortus* ^a^	5	2	1	2	0	0
Viridiplantae	Anthophyta	Monocotyledonae	Poales	Poaceae	*Leymus*		2	0	0	0	2	0
Viridiplantae	Anthophyta	Monocotyledonae	Poales	Poaceae	*Neyraudia*	*reynaudiana*	1	1	0	0	0	0
Viridiplantae	Anthophyta	Monocotyledonae	Poales	Poaceae	*Panicum*		25	6	4	8	0	7
Viridiplantae	Anthophyta	Monocotyledonae	Poales	Poaceae	*Panicum*	*coloratum*	1	0	0	0	1	0
Viridiplantae	Anthophyta	Monocotyledonae	Poales	Poaceae	*Perotis*	*hordeiformis*	12	2	3	4	0	3
Viridiplantae	Anthophyta	Monocotyledonae	Poales	Poaceae	*Poa*	*infirma*	1	0	0	0	1	0
Viridiplantae	Anthophyta	Monocotyledonae	Poales	Poaceae	*Setaria*		1	0	0	1	0	0
Viridiplantae	Anthophyta	Monocotyledonae	Poales	Poaceae	*Sporobolus*	*carolii* ^a^	7	1	0	3	0	3
Viridiplantae	Anthophyta	Monocotyledonae	Poales	Poaceae	*Themeda*	*triandra* ^a^	2	0	0	1	0	1
Viridiplantae	Anthophyta	Monocotyledonae	Poales	Poaceae	*Thyridolepis*	*mitchelliana* ^a^	33	6	7	8	4	8
Viridiplantae	Anthophyta	Monocotyledonae	Poales	Poaceae	*Tripogon*	*loliiformis* ^a^	7	0	1	4	0	2
Viridiplantae	Anthophyta	Monocotyledonae	Poales	Poaceae	*Willkommia*	*texana*	11	3	2	5	0	1
Viridiplantae	Coniferophyta	Pinopsida	Pinales	Cupressaceae	*Callitris*	*columellaris* ^a^	5	0	2	0	0	3
Viridiplantae	Coniferophyta	Pinopsida	Pinales	Taxodiaceae	*Cryptomeria*	*japonica*	2	0	0	1	1	0

^a^ Native species.

**TABLE A4:** Abundance (or RRA) of taxa in the diet of the northern hairy–nosed wombat (NHNW) at Epping Forest National Park (EFNP) throughout each sampling period from 2013 to 2021.

Taxon							Season								
Kingdom	Phylum	Class	Order	Family	Genus	Species	2020/21 Mean	Winter 2020 Mean	Spring 2020 Mean	Summer 2020/21 Mean	Winter 2021 Mean	2013/17 Mean	Autumn 2013	Autumn 2014	Autumn 2017
Unit							(%)	(%)	(%)	(%)	(%)	(%)	(%)	(%)	(%)
Unassigned							0.39263	0.46739	0.29588	0.25430	0.62798	0.12367	0.05110	0.02853	0.29137
Fungi							0.00019		0.00056						
Fungi	Ascomycota	Dothideomycetes	Pleosporales	Didymellaceae	*Didymella*		0.00006				0.00026				
Fungi	Ascomycota	Dothideomycetes	Pleosporales	Didymellaceae	*Epicoccum*	*nigrum*	0.00071		0.00214						
Fungi	Ascomycota	Dothideomycetes	Pleosporales	Sporormiaceae	*Preussia*		0.00091				0.00409	0.00612			0.01835
Fungi	Ascomycota	Sordariomycetes	Hypocreales	Hypocreaceae	*Trichoderma*		0.00010				0.00046				
Fungi	Ascomycota	Sordariomycetes	Hypocreales	Incertae sedis	*Acremonium*	*persicinum*						0.00160	0.00479		
Fungi	Basidiomycota	Cystobasidiomycetes	Cystobasidiales	Cystobasidiaceae			0.00012				0.00054				
Fungi	Basidiomycota	Cystobasidiomycetes	Cystobasidiales	Cystobasidiaceae	*Cystobasidium*	*benthicum*	0.00159		0.00019		0.00688				
Fungi	Basidiomycota	Tremellomycetes	Filobasidiales	Filobasidiaceae	*Naganishia*	*albida*	0.00007				0.00031				
Fungi	Basidiomycota	Tremellomycetes	Filobasidiales	Piskurozymaceae	*Solicoccozyma*	*phenolica*	0.00009				0.00039				
Viridiplantae							0.00007				0.00032				
Viridiplantae	Anthophyta	Eudicotyledonae					0.00780	0.00016		0.00535	0.02893				
Viridiplantae	Anthophyta	Eudicotyledonae	Asterales	Asteraceae			0.00613	0.00279	0.01676						
Viridiplantae	Anthophyta	Eudicotyledonae	Asterales	Asteraceae	*Pterocaulon*	*sphacelatum* ^a^	0.14315				0.64416				
Viridiplantae	Anthophyta	Eudicotyledonae	Asterales	Asteraceae	*Taraxacum*		0.00026		0.00077						
Viridiplantae	Anthophyta	Eudicotyledonae	Asterales	Asteraceae	*Vittadinia*	*dissecta* ^a^	0.00123		0.00369						
Viridiplantae	Anthophyta	Eudicotyledonae	Brassicales	Cleomaceae	*Cleome*	*cleomoides* ^a^	0.02068			0.08271					
Viridiplantae	Anthophyta	Eudicotyledonae	Caryophyllales	Aizoaceae	*Trianthema*		0.00252				0.01132				
Viridiplantae	Anthophyta	Eudicotyledonae	Caryophyllales	Amaranthaceae	*Alternanthera*	*sessilis*	0.00052			0.00208					
Viridiplantae	Anthophyta	Eudicotyledonae	Caryophyllales	Amaranthaceae	*Salsola*		0.00105				0.00471				
Viridiplantae	Anthophyta	Eudicotyledonae	Caryophyllales	Molluginaceae	*Hypertelis*		0.00138			0.00552					
Viridiplantae	Anthophyta	Eudicotyledonae	Caryophyllales	Nyctaginaceae	*Boerhavia*	*erecta*	4.15776	0.18958	1.96118	13.86565	0.00343	0.06922		0.20767	
Viridiplantae	Anthophyta	Eudicotyledonae	Caryophyllales	Portulacaceae	*Portulaca*		0.02046			0.07756	0.00480				
Viridiplantae	Anthophyta	Eudicotyledonae	Caryophyllales	Portulacaceae	*Portulaca*	*oleracea*	0.03118			0.03608	0.09973				
Viridiplantae	Anthophyta	Eudicotyledonae	Celastrales	Celastraceae	*Denhamia*	*celastroides* ^a^	0.00030			0.00120					
Viridiplantae	Anthophyta	Eudicotyledonae	Cucurbitales	Cucurbitaceae	*Cucumis*	*sativus*	0.00331				0.01488				
Viridiplantae	Anthophyta	Eudicotyledonae	Fabales	Fabaceae			0.00173		0.00519						
Viridiplantae	Anthophyta	Eudicotyledonae	Fabales	Fabaceae	*Acacia*		0.01327	0.03163	0.01610	0.00498	0.00229				
Viridiplantae	Anthophyta	Eudicotyledonae	Fabales	Fabaceae	*Acacia*	*ampliceps* ^a^	0.01076	0.00400	0.02996						
Viridiplantae	Anthophyta	Eudicotyledonae	Fabales	Fabaceae	*Acacia*	*confusa*	0.00894	0.04598							
Viridiplantae	Anthophyta	Eudicotyledonae	Fabales	Fabaceae	*Bauhinia*	*apertilobata* ^a^	0.00835	0.00892	0.01901	0.00110					
Viridiplantae	Anthophyta	Eudicotyledonae	Fabales	Fabaceae	*Chamaecrista*		0.00223	0.00259			0.00776	0.00713		0.02140	
Viridiplantae	Anthophyta	Eudicotyledonae	Fabales	Fabaceae	*Desmodium*		0.00797			0.03165	0.00026	0.00819		0.02457	
Viridiplantae	Anthophyta	Eudicotyledonae	Fabales	Fabaceae	*Desmodium*	*triflorum*	0.00017			0.00069					
Viridiplantae	Anthophyta	Eudicotyledonae	Fabales	Fabaceae	*Galactia*	*striata*	0.01215			0.04266	0.00670	0.08345			0.25036
Viridiplantae	Anthophyta	Eudicotyledonae	Fabales	Fabaceae	*Glycine*	*clandestina* ^a^						0.05152		0.15456	
Viridiplantae	Anthophyta	Eudicotyledonae	Fabales	Fabaceae	*Indigofera*	*astragalina* ^a^	0.00170				0.00763	0.00396		0.01189	
Viridiplantae	Anthophyta	Eudicotyledonae	Fabales	Fabaceae	*Indigofera*	*colutea*	0.11448	0.00400		0.42806	0.03008	0.09010		0.27029	
Viridiplantae	Anthophyta	Eudicotyledonae	Fabales	Fabaceae	*Indigofera*	*linifolia* ^a^	0.09413			0.37650		0.00766		0.02299	
Viridiplantae	Anthophyta	Eudicotyledonae	Fabales	Fabaceae	*Indigofera*	*linnaei* ^a^	0.00124			0.00496					
Viridiplantae	Anthophyta	Eudicotyledonae	Fabales	Fabaceae	*Neptunia*	*monosperma* ^a^	0.00093	0.00478							
Viridiplantae	Anthophyta	Eudicotyledonae	Fabales	Fabaceae	*Rhynchosia*	*minima* ^a^	0.00221				0.00992	0.22375			0.67124
Viridiplantae	Anthophyta	Eudicotyledonae	Fabales	Fabaceae	*Tephrosia*		0.00074			0.00296					
Viridiplantae	Anthophyta	Eudicotyledonae	Fabales	Fabaceae	*Zornia*	*gibbosa*	0.02706			0.10826					
Viridiplantae	Anthophyta	Eudicotyledonae	Fagales	Juglandaceae	*Juglans*	*nigra*	0.00695		0.02085						
Viridiplantae	Anthophyta	Eudicotyledonae	Gentianales	Apocynaceae			0.00048			0.00190					
Viridiplantae	Anthophyta	Eudicotyledonae	Gentianales	Apocynaceae	*Alstonia*	*scholaris* ^a^	0.00383				0.01723				
Viridiplantae	Anthophyta	Eudicotyledonae	Lamiales	Acanthaceae	*Justicia*	*procumbens*	0.00669			0.02676					
Viridiplantae	Anthophyta	Eudicotyledonae	Lamiales	Acanthaceae	*Ruellia*	*patula*	0.00153				0.00688				
Viridiplantae	Anthophyta	Eudicotyledonae	Lamiales	Lamiaceae			0.00076	0.00389							
Viridiplantae	Anthophyta	Eudicotyledonae	Lamiales	Oleaceae	*Jasminum*	*fluminense*						0.00661		0.01982	
Viridiplantae	Anthophyta	Eudicotyledonae	Malpighiales	Euphorbiaceae	*Euphorbia*	*humifusa*	0.00051	0.00207		0.00042					
Viridiplantae	Anthophyta	Eudicotyledonae	Malpighiales	Phyllanthaceae	*Phyllanthus*	*ussuriensis*	0.01135	0.00754		0.00869	0.03472				
Viridiplantae	Anthophyta	Eudicotyledonae	Malpighiales	Phyllanthaceae	*Synostemon*		0.01224		0.00375	0.00064	0.04875				
Viridiplantae	Anthophyta	Eudicotyledonae	Malvales	Malvaceae			0.34555	0.10792	0.04189	0.18638	1.18804	0.11660		0.13396	0.21583
Viridiplantae	Anthophyta	Eudicotyledonae	Malvales	Malvaceae	*Abutilon*	*indicum* ^a^	0.00008		0.00023						
Viridiplantae	Anthophyta	Eudicotyledonae	Malvales	Malvaceae	*Hibiscus*		0.01355	0.02483	0.02420	0.00232	0.00032				
Viridiplantae	Anthophyta	Eudicotyledonae	Malvales	Malvaceae	*Hibiscus*	*micranthus*	0.00119	0.00610							
Viridiplantae	Anthophyta	Eudicotyledonae	Malvales	Malvaceae	*Malvastrum*	*coromandelianum*	0.09459	0.07391			0.36100				
Viridiplantae	Anthophyta	Eudicotyledonae	Malvales	Malvaceae	*Trochetiopsis*	*erythroxylon*	0.34014	0.35860	0.25091	0.74435	0.00309				
Viridiplantae	Anthophyta	Eudicotyledonae	Malvales	Malvaceae	*Waltheria*	*indica*	0.34479	0.38668	0.03224	0.89201	0.16133	0.30754		0.92263	
Viridiplantae	Anthophyta	Eudicotyledonae	Myrtales	Myrtaceae			0.00716		0.02149						
Viridiplantae	Anthophyta	Eudicotyledonae	Myrtales	Myrtaceae	*Angophora*	*bakeri* ^a^	0.01246	0.00358	0.02363	0.01553					
Viridiplantae	Anthophyta	Eudicotyledonae	Myrtales	Myrtaceae	*Corymbia*	*ficifolia* ^a^	0.01775		0.03193	0.02844					
Viridiplantae	Anthophyta	Eudicotyledonae	Myrtales	Myrtaceae	*Eucalyptus*		0.00405		0.00953	0.00351					
Viridiplantae	Anthophyta	Eudicotyledonae	Myrtales	Myrtaceae	*Eucalyptus*	*brachyandra* ^a^	0.02770	0.01981	0.05426	0.02305					
Viridiplantae	Anthophyta	Eudicotyledonae	Myrtales	Myrtaceae	*Melaleuca*	*armillaris* ^a^	0.00587	0.03017							
Viridiplantae	Anthophyta	Eudicotyledonae	Myrtales	Myrtaceae	*Melaleuca*	*quinquenervia* ^a^	0.00042				0.00188				
Viridiplantae	Anthophyta	Eudicotyledonae	Oxalidales	Oxalidaceae	*Oxalis*	*corniculata*	0.02746				0.12358				
Viridiplantae	Anthophyta	Eudicotyledonae	Rosales	Rhamnaceae	*Ventilago*	*denticulata*	0.00214			0.00857					
Viridiplantae	Anthophyta	Eudicotyledonae	Sapindales	Meliaceae			0.00018			0.00072					
Viridiplantae	Anthophyta	Eudicotyledonae	Sapindales	Rutaceae	*Geijera*	*parviflora* ^a^	0.00264		0.00792						
Viridiplantae	Anthophyta	Eudicotyledonae	Sapindales	Rutaceae			0.00110				0.00496				
Viridiplantae	Anthophyta	Eudicotyledonae	Sapindales	Sapindaceae	*Alectryon*	*excelsus*	0.00297		0.00892						
Viridiplantae	Anthophyta	Eudicotyledonae	Solanales	Convolvulaceae	*Evolvulus*	*alsinoides* ^a^	1.31961	0.14312	1.32908	2.46839	1.04246	0.04729		0.14188	
Viridiplantae	Anthophyta	Eudicotyledonae	Solanales	Convolvulaceae	*Ipomoea*		0.00490			0.01958					
Viridiplantae	Anthophyta	Eudicotyledonae	Solanales	Convolvulaceae	*Ipomoea*	*oenotherae*	0.00316			0.01263		0.10040		0.30120	
Viridiplantae	Anthophyta	Eudicotyledonae	Solanales	Convolvulaceae	*Ipomoea*	*polymorpha* ^a^	0.04394			0.17578		0.14215		0.42644	
Viridiplantae	Anthophyta	Eudicotyledonae	Solanales	Solanaceae	*Solanum*	*dianthophorum* ^a^	0.00318	0.00222	0.00824						
Viridiplantae	Anthophyta	Eudicotyledonae	Solanales	Solanaceae			0.00039	0.00202							
Viridiplantae	Anthophyta	Eudicotyledonae	Vitales	Vitaceae	*Vitis*	*heyneana*	0.00504	0.00447	0.00229		0.01531				
Viridiplantae	Anthophyta	Eudicotyledonae	Zygophyllales	Zygophyllaceae	*Tribulus*		1.08365		0.01110	4.31982		0.13554		0.40662	
Viridiplantae	Anthophyta	Eudicotyledonae	Zygophyllales	Zygophyllaceae	*Tribulus*	*pentandrus*	0.09384			0.37535		0.02325		0.06975	
Viridiplantae	Anthophyta	Eudicotyledonae	Zygophyllales	Zygophyllaceae	*Tribulus*	*terrestris*	0.00567			0.02268					
Viridiplantae	Anthophyta	Monocotyledonae	Poales	Cyperaceae	*Bulbostylis*	*barbata* ^a^	0.00568			0.02273					
Viridiplantae	Anthophyta	Monocotyledonae	Poales	Cyperaceae	*Cyperus*		0.00023			0.00092					
Viridiplantae	Anthophyta	Monocotyledonae	Poales	Cyperaceae	*Fimbristylis*		2.10009	1.01266	1.59927	5.00480	0.53502	0.77545	1.23436	0.64521	0.44677
Viridiplantae	Anthophyta	Monocotyledonae	Poales	Cyperaceae	*Fimbristylis*	*dichotoma* ^a^	0.00872		0.00120	0.01891	0.01618				
Viridiplantae	Anthophyta	Monocotyledonae	Poales	Cyperaceae	*Fimbristylis*	*stauntonii*	0.00261			0.01045					
Viridiplantae	Anthophyta	Monocotyledonae	Poales	Poaceae			0.26475	0.02426	0.05968	0.59075	0.41606	0.98732	1.81322	1.06134	0.08741
Viridiplantae	Anthophyta	Monocotyledonae	Poales	Poaceae	*Acrachne*	*racemosa* ^a^	0.00013			0.00053					
Viridiplantae	Anthophyta	Monocotyledonae	Poales	Poaceae	*Aristida*		0.03097	0.00870	0.01222	0.06726	0.03779	0.15062	0.06707	0.12365	0.26116
Viridiplantae	Anthophyta	Monocotyledonae	Poales	Poaceae	*Aristida*	*calycina* ^a^	0.03450	0.01044	0.01604	0.09838	0.01137	0.20423	0.54373	0.06896	
Viridiplantae	Anthophyta	Monocotyledonae	Poales	Poaceae	*Aristida*	*caput‐medusae* ^a^	0.10153	0.03032	0.00469	0.31934	0.06409	0.38132	0.61159	0.40504	0.12734
Viridiplantae	Anthophyta	Monocotyledonae	Poales	Poaceae	*Aristida*	*holathera* ^a^	0.00150			0.00598					
Viridiplantae	Anthophyta	Monocotyledonae	Poales	Poaceae	*Aristida*	*ingrata* ^a^	0.05862	0.00183	0.00227	0.12528	0.11786	0.06699	0.05828	0.14267	
Viridiplantae	Anthophyta	Monocotyledonae	Poales	Poaceae	*Arundinella*	*bengalensis*	0.01295	0.01238	0.02784	0.00421	0.00093	0.00766		0.02299	
Viridiplantae	Anthophyta	Monocotyledonae	Poales	Poaceae	*Avena*	*ventricosa*	0.00057				0.00254				
Viridiplantae	Anthophyta	Monocotyledonae	Poales	Poaceae	*Bothriochloa*		0.01767	0.03470	0.01095	0.02911		0.35549	0.92617		0.14029
Viridiplantae	Anthophyta	Monocotyledonae	Poales	Poaceae	*Bothriochloa*	*pertusa*	0.03179	0.09053	0.02646	0.00927	0.01374	0.13816		0.00872	0.40576
Viridiplantae	Anthophyta	Monocotyledonae	Poales	Poaceae	*Brachiaria*		0.00196	0.00776		0.00182					
Viridiplantae	Anthophyta	Monocotyledonae	Poales	Poaceae	*Brachiaria*	*ramosa*	0.01348	0.06930							
Viridiplantae	Anthophyta	Monocotyledonae	Poales	Poaceae	*Brachiaria*	*subquadripara*	2.69515	0.06022	0.06234	10.65065		7.16770	0.25550	20.56341	0.68418
Viridiplantae	Anthophyta	Monocotyledonae	Poales	Poaceae	*Cenchrus*	*ciliaris*	71.57183	92.56205	75.95207	30.80976	92.49235	40.83702	59.50003	31.05397	31.95705
Viridiplantae	Anthophyta	Monocotyledonae	Poales	Poaceae	*Chloris*	*pumilio* ^a^	0.00757			0.03027					
Viridiplantae	Anthophyta	Monocotyledonae	Poales	Poaceae	*Chrysopogon*		0.15951	0.04786	0.34529	0.13547	0.00560	0.25155	0.13733	0.11335	0.50397
Viridiplantae	Anthophyta	Monocotyledonae	Poales	Poaceae	*Chrysopogon*	*latifolius* ^a^	10.73240	3.73528	17.46458	16.48794	0.28162	22.10596	13.78875	16.08817	36.44094
Viridiplantae	Anthophyta	Monocotyledonae	Poales	Poaceae	*Dactyloctenium*	*aegyptium*	0.06913			0.27653		0.00661		0.01982	
Viridiplantae	Anthophyta	Monocotyledonae	Poales	Poaceae	*Dinebra*		0.00281			0.01125					
Viridiplantae	Anthophyta	Monocotyledonae	Poales	Poaceae	*Enneapogon*	*scoparius*	0.39719	0.27067	0.09870	0.62217	0.70252	10.48575	14.82032	0.59527	16.04166
Viridiplantae	Anthophyta	Monocotyledonae	Poales	Poaceae	*Enteropogon*	*ramosus* ^a^	0.00230	0.00793		0.00302		0.34857	0.25470		0.79102
Viridiplantae	Anthophyta	Monocotyledonae	Poales	Poaceae	*Eragrostis*		0.13987		0.00011	0.55935		0.05073		0.15219	
Viridiplantae	Anthophyta	Monocotyledonae	Poales	Poaceae	*Eragrostis*	*aegyptiaca*	0.00018			0.00071					
Viridiplantae	Anthophyta	Monocotyledonae	Poales	Poaceae	*Eragrostis*	*andicola*	0.00006			0.00023					
Viridiplantae	Anthophyta	Monocotyledonae	Poales	Poaceae	*Eragrostis*	*ciliaris*	0.00107			0.00429					
Viridiplantae	Anthophyta	Monocotyledonae	Poales	Poaceae	*Eragrostis*	*confertiflora* ^a^	0.00158			0.00632					
Viridiplantae	Anthophyta	Monocotyledonae	Poales	Poaceae	*Eragrostis*	*refracta*	0.32981			1.31924		0.02431		0.07292	
Viridiplantae	Anthophyta	Monocotyledonae	Poales	Poaceae	*Eriachne*		0.00079			0.00315					
Viridiplantae	Anthophyta	Monocotyledonae	Poales	Poaceae	*Eulalia*	*aurea* ^a^	0.00470		0.01250	0.00215		0.10311	0.08703		0.22231
Viridiplantae	Anthophyta	Monocotyledonae	Poales	Poaceae	*Heteropogon*	*contortus* ^a^	0.16618	0.06945	0.03702	0.26185	0.33693	9.15207	5.02128	13.78873	8.64620
Viridiplantae	Anthophyta	Monocotyledonae	Poales	Poaceae	*Melinis*	*repens*	0.00832				0.03746	2.61742		7.50153	0.35073
Viridiplantae	Anthophyta	Monocotyledonae	Poales	Poaceae	*Panicum*							0.19721	0.59163		
Viridiplantae	Anthophyta	Monocotyledonae	Poales	Poaceae	*Perotis*	*hordeiformis*	2.02493	0.00176	0.02628	7.73153	0.37326	1.82739	0.16447	3.98063	1.33707
Viridiplantae	Anthophyta	Monocotyledonae	Poales	Poaceae	*Setaria*	*viridis*	0.00310			0.01241		0.12365		0.37095	
Viridiplantae	Anthophyta	Monocotyledonae	Poales	Poaceae	*Sporobolus*	*carolii* ^a^	0.00343			0.01371					
Viridiplantae	Anthophyta	Monocotyledonae	Poales	Poaceae	*Themeda*	*triandra* ^a^	0.00864			0.02403	0.01185				
Viridiplantae	Anthophyta	Monocotyledonae	Poales	Poaceae	*Themeda*	*villosa*						0.04981		0.04042	0.10899
Viridiplantae	Anthophyta	Monocotyledonae	Poales	Poaceae	*Thyridolepis*	*mitchelliana* ^a^	0.00006				0.00026				
Viridiplantae	Anthophyta	Monocotyledonae	Poales	Poaceae	*Tripogon*	*loliiformis* ^a^	0.00280	0.00316	0.00603	0.00072		0.02289	0.06866		
Viridiplantae	Anthophyta	Monocotyledonae	Poales	Poaceae	*Willkommia*	*texana*	0.01300			0.05199		0.00106		0.00317	
Viridiplantae	Anthophyta	Monocotyledonae	Zingiberales	Musaceae	*Musa*	*mannii* ^a^						0.00423		0.01268	
Viridiplantae	Coniferophyta	Pinopsida	Pinales	Pinaceae	*Pinus*	*hartwegii*	0.00114		0.00061	0.00147	0.00257				

^a^ Native species.

**TABLE A5:** Abundance (or RRA) of taxa in the diet of the northern hairy‐nosed wombat (NHNW) at Richard Underwood Nature Refuge (RUNR) throughout each sampling period from 2020 to 2021.

Taxon							Season					
Kingdom	Phylum	Class	Order	Family	Genus	Species	2020/21 Mean	Winter 2020 Mean	Summer 2020/21 Mean	Autumn 2021 Mean	Winter 2021 Mean	Spring 2021 Mean
Unit							(%)	(%)	(%)	(%)	(%)	(%)
Unassigned							0.15291	0.06377	0.21542	0.02506	0.08329	0.35475
Fungi							0.00009		0.00041			
Fungi	Ascomycota	Dothideomycetes	Capnodiales	Cladosporiaceae	*Cladosporium*		0.00005			0.00025		
Fungi	Ascomycota	Dothideomycetes	Pleosporales	Didymellaceae	*Epicoccum*		0.00006	0.00040				
Fungi	Ascomycota	Dothideomycetes	Pleosporales	Sporormiaceae	*Preussia*		0.00534		0.02538			
Fungi	Ascomycota	Leotiomycetes	Helotiales	Helotiaceae	*Acidea*	*extrema*	0.00016			0.00074		
Fungi	Ascomycota	Leotiomycetes	Helotiales	Hyaloscyphaceae			0.00005				0.00022	
Fungi	Ascomycota	Sordariomycetes	Hypocreales	Hypocreaceae	*Trichoderma*		0.00006					0.00029
Fungi	Basidiomycota	Agaricomycetes					0.00564		0.00407			0.02271
Fungi	Basidiomycota	Agaricomycetes	Phallales	Clathraceae	*Ileodictyon*	*gracile*	0.00027					0.00126
Fungi	Basidiomycota	Agaricomycetes	Polyporales	Phanerochaetaceae	*Oxychaete*		0.00004				0.00019	
Fungi	Basidiomycota	Agaricomycetes	Russulales	Peniophoraceae	*Peniophora*		0.00020				0.00096	
Fungi	Basidiomycota	Cystobasidiomycetes	Cystobasidiales	Cystobasidiaceae			0.00020					0.00094
Fungi	Basidiomycota	Cystobasidiomycetes	Cystobasidiales	Cystobasidiaceae	*Cystobasidium*	*benthicum*	0.00057		0.00270			
Fungi	Basidiomycota	Tremellomycetes	Filobasidiales	Filobasidiaceae	*Naganishia*	*albida*	0.00105		0.00497			
Viridiplantae	Anthophyta	Eudicotyledonae					0.00414	0.00266		0.00241	0.00101	0.01427
Viridiplantae	Anthophyta	Eudicotyledonae	Asterales	Asteraceae			0.01911	0.01343	0.00026	0.04023		0.04019
Viridiplantae	Anthophyta	Eudicotyledonae	Asterales	Asteraceae	*Calotis*		0.02681	0.08384	0.01929	0.03271		0.01246
Viridiplantae	Anthophyta	Eudicotyledonae	Asterales	Asteraceae	*Lactuca*	*virosa* ^a^	0.00160					0.00758
Viridiplantae	Anthophyta	Eudicotyledonae	Asterales	Asteraceae	*Podolepis*		0.00091					0.00432
Viridiplantae	Anthophyta	Eudicotyledonae	Asterales	Asteraceae	*Podolepis*	*robusta* ^a^	0.00170					0.00806
Viridiplantae	Anthophyta	Eudicotyledonae	Asterales	Asteraceae	*Pterocaulon*	*sphacelatum* ^a^	0.00328	0.02075				
Viridiplantae	Anthophyta	Eudicotyledonae	Asterales	Asteraceae	*Vittadinia*	*dissecta* ^a^	0.00291	0.00367		0.01106		
Viridiplantae	Anthophyta	Eudicotyledonae	Brassicales	Brassicaceae			0.00083	0.00472			0.00038	
Viridiplantae	Anthophyta	Eudicotyledonae	Brassicales	Brassicaceae	*Brassica*	*carinata*	0.00389				0.00978	0.00868
Viridiplantae	Anthophyta	Eudicotyledonae	Caryophyllales	Amaranthaceae			0.00324					0.01537
Viridiplantae	Anthophyta	Eudicotyledonae	Caryophyllales	Amaranthaceae	*Alternanthera*		0.00630	0.03992				
Viridiplantae	Anthophyta	Eudicotyledonae	Caryophyllales	Amaranthaceae	*Alternanthera*	*sessilis*	0.21085	0.73128	0.01500	0.43808		
Viridiplantae	Anthophyta	Eudicotyledonae	Caryophyllales	Amaranthaceae	*Chenopodium*		0.09907	0.04539	0.01300	0.12664	0.07699	0.21990
Viridiplantae	Anthophyta	Eudicotyledonae	Caryophyllales	Amaranthaceae	*Chenopodium*	*opulifolium*	0.00133					0.00631
Viridiplantae	Anthophyta	Eudicotyledonae	Caryophyllales	Amaranthaceae	*Dysphania*		0.03846	0.20812		0.01857		0.00805
Viridiplantae	Anthophyta	Eudicotyledonae	Caryophyllales	Amaranthaceae	*Ptilotus*	*polystachyus* ^a^	0.00918	0.01988		0.02872		
Viridiplantae	Anthophyta	Eudicotyledonae	Caryophyllales	Nyctaginaceae	*Boerhavia*	*Erecta*	2.27001	4.87653	5.22307	1.74566	0.02110	0.13534
Viridiplantae	Anthophyta	Eudicotyledonae	Caryophyllales	Portulacaceae	*Portulaca*		0.07220	0.43525		0.01649		
Viridiplantae	Anthophyta	Eudicotyledonae	Caryophyllales	Portulacaceae	*Portulaca*	*Oleracea*	0.00136	0.00864				
Viridiplantae	Anthophyta	Eudicotyledonae	Cucurbitales	Cucurbitaceae	*Cucumis*	*Sativus*	0.00807		0.00806		0.02884	0.00143
Viridiplantae	Anthophyta	Eudicotyledonae	Ericales	Theaceae	*Camellia*		0.00006				0.00027	
Viridiplantae	Anthophyta	Eudicotyledonae	Fabales	Fabaceae	*Acacia*	*confusa*	0.02448	0.00993	0.04444	0.00847	0.00983	0.04610
Viridiplantae	Anthophyta	Eudicotyledonae	Fabales	Fabaceae	*Glycine*	*clandestina* ^a^	0.08500	0.38160	0.01840	0.07372	0.02304	0.00241
Viridiplantae	Anthophyta	Eudicotyledonae	Fabales	Fabaceae	*Medicago*	*minima*	0.00011					0.00052
Viridiplantae	Anthophyta	Eudicotyledonae	Gentianales	Apocynaceae	*Parsonsia*	*crebriflora* ^a^	0.00017					0.00080
Viridiplantae	Anthophyta	Eudicotyledonae	Geraniales	Geraniaceae	*Erodium*		0.00163					0.00776
Viridiplantae	Anthophyta	Eudicotyledonae	Lamiales				0.01739	0.01428	0.04332	0.00991		0.01867
Viridiplantae	Anthophyta	Eudicotyledonae	Lamiales	Acanthaceae	*Justicia*	*procumbens*	0.00524			0.02488		
Viridiplantae	Anthophyta	Eudicotyledonae	Lamiales	Lamiaceae			0.00643	0.00371	0.01656			0.01120
Viridiplantae	Anthophyta	Eudicotyledonae	Lamiales	Oleaceae	*Fraxinus*	*velutina*	0.00133		0.00632			
Viridiplantae	Anthophyta	Eudicotyledonae	Lamiales	Oleaceae	*Jasminum*	*fluminense*	0.00575		0.00127	0.01791	0.00815	
Viridiplantae	Anthophyta	Eudicotyledonae	Lamiales	Scrophulariaceae	*Myoporum*	*insulare* ^a^	0.00085		0.00403			
Viridiplantae	Anthophyta	Eudicotyledonae	Malpighiales	Euphorbiaceae	*Euphorbia*	*humifusa*	0.00115			0.00482		0.00066
Viridiplantae	Anthophyta	Eudicotyledonae	Malpighiales	Phyllanthaceae	*Phyllanthus*	*ussuriensis*	0.00401			0.01290		0.00616
Viridiplantae	Anthophyta	Eudicotyledonae	Malpighiales	Phyllanthaceae	*Synostemon*		0.00022			0.00104		
Viridiplantae	Anthophyta	Eudicotyledonae	Malvales	Malvaceae			0.24230	0.26128	0.16672	0.27702	0.04009	0.47115
Viridiplantae	Anthophyta	Eudicotyledonae	Malvales	Malvaceae	*Abutilon*	*indicum* ^a^	0.50938	0.25979	0.08827	1.68459	0.03207	0.41977
Viridiplantae	Anthophyta	Eudicotyledonae	Malvales	Malvaceae	*Hibiscus*		0.69795	0.25237	1.11028	0.28576	1.35184	0.37810
Viridiplantae	Anthophyta	Eudicotyledonae	Malvales	Malvaceae	*Hibiscus*	*micranthus*	0.02109	0.00710	0.05322	0.00453	0.03174	0.00538
Viridiplantae	Anthophyta	Eudicotyledonae	Malvales	Malvaceae	*Malvastrum*	*coromandelianum*	1.11390	1.13019	0.01757	3.60801	0.23432	0.58349
Viridiplantae	Anthophyta	Eudicotyledonae	Malvales	Malvaceae	*Trochetiopsis*	*erythroxylon*	0.01146		0.00830	0.00080		0.04533
Viridiplantae	Anthophyta	Eudicotyledonae	Myrtales	Myrtaceae	*Angophora*	*bakeri* ^a^	0.00530	0.01502	0.00609	0.00780		
Viridiplantae	Anthophyta	Eudicotyledonae	Myrtales	Myrtaceae	*Angophora*	*costata* ^a^	0.00038		0.00182			
Viridiplantae	Anthophyta	Eudicotyledonae	Myrtales	Myrtaceae	*Corymbia*	*ficifolia* ^a^	0.01316		0.06252			
Viridiplantae	Anthophyta	Eudicotyledonae	Myrtales	Myrtaceae	*Eucalyptus*		0.02901	0.05787	0.07765	0.00562	0.01110	
Viridiplantae	Anthophyta	Eudicotyledonae	Myrtales	Myrtaceae	*Eucalyptus*	*brachyandra* ^a^	0.04494	0.07636	0.15548			0.00074
Viridiplantae	Anthophyta	Eudicotyledonae	Oxalidales	Oxalidaceae	*Oxalis*	*corniculata*	0.05187			0.01155	0.00663	0.22821
Viridiplantae	Anthophyta	Eudicotyledonae	Proteales	Proteaceae	*Grevillea*	*robusta* ^a^	0.00086				0.00408	
Viridiplantae	Anthophyta	Eudicotyledonae	Rosales	Urticaceae			0.00067		0.00317			
Viridiplantae	Anthophyta	Eudicotyledonae	Rosales	Urticaceae	*Urtica*		0.00782		0.03715			
Viridiplantae	Anthophyta	Eudicotyledonae	Sapindales	Rutaceae	*Citrus*	*reticulata*	0.00041				0.00196	
Viridiplantae	Anthophyta	Eudicotyledonae	Sapindales	Rutaceae	*Geijera*	*parviflora* ^a^	0.08890	0.05397	0.01364	0.29292	0.01834	0.05689
Viridiplantae	Anthophyta	Eudicotyledonae	Sapindales	Sapindaceae	*Alectryon*	*excelsus*	0.02107	0.01329	0.07429			0.01583
Viridiplantae	Anthophyta	Eudicotyledonae	Saxifragales	Crassulaceae	*Crassula*	*campestris*	0.03613	0.00417				0.16847
Viridiplantae	Anthophyta	Eudicotyledonae	Saxifragales	Haloragaceae	*Haloragis*	*erecta*	0.00276			0.01313		
Viridiplantae	Anthophyta	Eudicotyledonae	Solanales	Convolvulaceae	*Evolvulus*	*alsinoides* ^a^	1.65370	0.23981	0.48754	3.49032	0.38610	3.31125
Viridiplantae	Anthophyta	Eudicotyledonae	Solanales	Solanaceae	*Capsicum*		0.00049				0.00231	
Viridiplantae	Anthophyta	Eudicotyledonae	Solanales	Solanaceae	*Solanum*		0.20481	0.05401		0.00422	0.00831	0.91981
Viridiplantae	Anthophyta	Eudicotyledonae	Solanales	Solanaceae	*Solanum*	*dianthophorum* ^a^	0.11065		0.00114	0.00230	0.01666	0.50548
Viridiplantae	Anthophyta	Eudicotyledonae	Solanales	Solanaceae	*Solanum*	*tuberosum*	0.00006		0.00028			
Viridiplantae	Anthophyta	Eudicotyledonae	Vitales	Vitaceae	*Vitis*	*heyneana*	0.00268	0.01352		0.00021		0.00237
Viridiplantae	Anthophyta	Monocotyledonae	Asparagales	Xanthorrhoeaceae	*Dianella*	*longifolia* ^a^	0.00113					0.00539
Viridiplantae	Anthophyta	Monocotyledonae	Poales	Cyperaceae	*Bulbostylis*	*barbata* ^a^	0.00088	0.00558				
Viridiplantae	Anthophyta	Monocotyledonae	Poales	Cyperaceae	*Cyperus*		0.06659	0.01740	0.19141	0.01665		0.09519
Viridiplantae	Anthophyta	Monocotyledonae	Poales	Cyperaceae	*Eleocharis*		0.00008			0.00040		
Viridiplantae	Anthophyta	Monocotyledonae	Poales	Cyperaceae	*Fimbristylis*		0.00134	0.00410	0.00236			0.00095
Viridiplantae	Anthophyta	Monocotyledonae	Poales	Poaceae			14.49403	7.41536	8.54884	20.93841	10.95397	22.84393
Viridiplantae	Anthophyta	Monocotyledonae	Poales	Poaceae	*Aristida*		0.76938	0.13131	1.73958	1.66590	0.04059	0.11002
Viridiplantae	Anthophyta	Monocotyledonae	Poales	Poaceae	*Aristida*	*calycina* ^a^	0.28758	0.03795	0.75838	0.52140		0.05777
Viridiplantae	Anthophyta	Monocotyledonae	Poales	Poaceae	*Aristida*	*caput‐medusae* ^a^	0.21729	0.05739	0.39790	0.56735	0.01249	0.01133
Viridiplantae	Anthophyta	Monocotyledonae	Poales	Poaceae	*Aristida*	*congesta*	0.00032			0.00151		
Viridiplantae	Anthophyta	Monocotyledonae	Poales	Poaceae	*Austrostipa*	*nodosa* ^a^	0.08745	0.37463	0.01179	0.06106	0.01990	0.04169
Viridiplantae	Anthophyta	Monocotyledonae	Poales	Poaceae	*Avena*	*ventricosa*	0.00220			0.00543	0.00286	0.00218
Viridiplantae	Anthophyta	Monocotyledonae	Poales	Poaceae	*Bothriochloa*	*ischaemum*	0.02243	0.03326		0.07365		0.00793
Viridiplantae	Anthophyta	Monocotyledonae	Poales	Poaceae	*Brachiaria*		0.00560			0.01934	0.00375	0.00349
Viridiplantae	Anthophyta	Monocotyledonae	Poales	Poaceae	*Brachiaria*	*ramosa*	0.25366	0.01985	0.12254	0.81984	0.23375	0.01385
Viridiplantae	Anthophyta	Monocotyledonae	Poales	Poaceae	*Brachiaria*	*subquadripara*	0.76313	4.73672	0.01753	0.05482		
Viridiplantae	Anthophyta	Monocotyledonae	Poales	Poaceae	*Cenchrus*		0.00155					0.00734
Viridiplantae	Anthophyta	Monocotyledonae	Poales	Poaceae	*Cenchrus*	*ciliaris*	68.63519	74.08396	78.46767	43.37675	86.14563	62.46412
Viridiplantae	Anthophyta	Monocotyledonae	Poales	Poaceae	*Chloris*		1.82794	0.12853		4.48406		4.10226
Viridiplantae	Anthophyta	Monocotyledonae	Poales	Poaceae	*Chloris*	*castilloniana*	0.00391			0.01142		0.00717
Viridiplantae	Anthophyta	Monocotyledonae	Poales	Poaceae	*Chloris*	*pumilio* ^a^	0.00171		0.00811			
Viridiplantae	Anthophyta	Monocotyledonae	Poales	Poaceae	*Chloris*	*ventricosa* ^a^	0.00088			0.00419		
Viridiplantae	Anthophyta	Monocotyledonae	Poales	Poaceae	*Chrysopogon*		0.02260	0.00203		0.10306	0.00278	
Viridiplantae	Anthophyta	Monocotyledonae	Poales	Poaceae	*Chrysopogon*	*latifolius* ^a^	0.22614	0.02069	0.01930	1.03935		
Viridiplantae	Anthophyta	Monocotyledonae	Poales	Poaceae	*Dactyloctenium*	*aegyptium*	0.00017	0.00107				
Viridiplantae	Anthophyta	Monocotyledonae	Poales	Poaceae	*Digitaria*		0.06958	0.00383	0.04913	0.04204	0.01946	0.21699
Viridiplantae	Anthophyta	Monocotyledonae	Poales	Poaceae	*Dinebra*		0.00195	0.00632		0.00453		
Viridiplantae	Anthophyta	Monocotyledonae	Poales	Poaceae	*Enneapogon*	*scoparius*	0.17979	0.08894	0.21620	0.55955	0.00216	0.00937
Viridiplantae	Anthophyta	Monocotyledonae	Poales	Poaceae	*Enteropogon*		0.00006			0.00029		
Viridiplantae	Anthophyta	Monocotyledonae	Poales	Poaceae	*Enteropogon*	*ramosus* ^a^	0.39267	1.10081	0.09513	0.87190	0.01569	0.05685
Viridiplantae	Anthophyta	Monocotyledonae	Poales	Poaceae	*Eragrostis*		0.01838	0.03477	0.00104	0.03915		0.02105
Viridiplantae	Anthophyta	Monocotyledonae	Poales	Poaceae	*Eragrostis*	*andicola*	0.00132	0.00836				
Viridiplantae	Anthophyta	Monocotyledonae	Poales	Poaceae	*Eragrostis*	*curvula*	0.65539	0.00881	0.14891	1.68323		1.27433
Viridiplantae	Anthophyta	Monocotyledonae	Poales	Poaceae	*Heteropogon*	*contortus* ^a^	0.04483	0.01023	0.00361	0.20164		
Viridiplantae	Anthophyta	Monocotyledonae	Poales	Poaceae	*Leymus*		0.00322				0.01528	
Viridiplantae	Anthophyta	Monocotyledonae	Poales	Poaceae	*Neyraudia*	*reynaudiana*	0.00016	0.00101				
Viridiplantae	Anthophyta	Monocotyledonae	Poales	Poaceae	*Panicum*		0.33920	0.06752	0.16581	1.24583		0.14891
Viridiplantae	Anthophyta	Monocotyledonae	Poales	Poaceae	*Panicum*	*coloratum*	0.00137				0.00650	
Viridiplantae	Anthophyta	Monocotyledonae	Poales	Poaceae	*Perotis*	*hordeiformis*	0.01474	0.01290	0.01298	0.02813		0.01922
Viridiplantae	Anthophyta	Monocotyledonae	Poales	Poaceae	*Poa*	*infirma*	0.00234				0.01110	
Viridiplantae	Anthophyta	Monocotyledonae	Poales	Poaceae	*Setaria*		0.00068			0.00322		
Viridiplantae	Anthophyta	Monocotyledonae	Poales	Poaceae	*Sporobolus*	*carolii* ^a^	0.04390	0.08737		0.08031		0.06269
Viridiplantae	Anthophyta	Monocotyledonae	Poales	Poaceae	*Themeda*	*triandra* ^a^	0.00111			0.00314		0.00211
Viridiplantae	Anthophyta	Monocotyledonae	Poales	Poaceae	*Thyridolepis*	*mitchelliana* ^a^	2.34837	2.07513	0.96851	8.21704	0.10282	0.31003
Viridiplantae	Anthophyta	Monocotyledonae	Poales	Poaceae	*Tripogon*	*loliiformis* ^a^	0.00936		0.00212	0.04128		0.00106
Viridiplantae	Anthophyta	Monocotyledonae	Poales	Poaceae	*Willkommia*	*texana*	0.19232	0.01764	0.01540	0.88291		0.00196
Viridiplantae	Coniferophyta	Pinopsida	Pinales	Cupressaceae	*Callitris*	*columellaris* ^a^	0.00764		0.00389			0.03240
Viridiplantae	Coniferophyta	Pinopsida	Pinales	Taxodiaceae	*Cryptomeria*	*japonica*	0.00069			0.00215	0.00113	

^a^ Native species.
